# The Impact of *ackA, pta*, and *ackA-pta* Mutations on Growth, Gene Expression and Protein Acetylation in *Escherichia coli* K-12

**DOI:** 10.3389/fmicb.2020.00233

**Published:** 2020-02-21

**Authors:** Andrea Schütze, Dirk Benndorf, Sebastian Püttker, Fabian Kohrs, Katja Bettenbrock

**Affiliations:** ^1^Max Planck Institute for Dynamics of Complex Technical Systems, Magdeburg, Germany; ^2^Bioprocess Engineering, Otto von Guericke University, Magdeburg, Germany

**Keywords:** acetate metabolism, overflow, acetyl-P, protein acetylation, fermentation

## Abstract

Acetate is a characteristic by-product of *Escherichia coli* K-12 growing in batch cultures with glucose, both under aerobic as well as anaerobic conditions. While the reason underlying aerobic acetate production is still under discussion, during anaerobic growth acetate production is important for ATP generation by substrate level phosphorylation. Under both conditions, acetate is produced by a pathway consisting of the enzyme phosphate acetyltransferase (Pta) producing acetyl-phosphate from acetyl-coenzyme A, and of the enzyme acetate kinase (AckA) producing acetate from acetyl-phosphate, a reaction that is coupled to the production of ATP. Mutants in the AckA-Pta pathway differ from each other in the potential to produce and accumulate acetyl-phosphate. In the publication at hand, we investigated different mutants in the acetate pathway, both under aerobic as well as anaerobic conditions. While under aerobic conditions only small changes in growth rate were observed, all acetate mutants showed severe reduction in growth rate and changes in the by-product pattern during anaerobic growth. The AckA^–^ mutant showed the most severe growth defect. The glucose uptake rate and the ATP concentration were strongly reduced in this strain. This mutant exhibited also changes in gene expression. In this strain, the *atoDAEB* operon was significantly upregulated under anaerobic conditions hinting to the production of acetoacetate. During anaerobic growth, protein acetylation increased significantly in the *ackA* mutant. Acetylation of several enzymes of glycolysis and central metabolism, of aspartate carbamoyl transferase, methionine synthase, catalase and of proteins involved in translation was increased. Supplementation of methionine and uracil eliminated the additional growth defect of the *ackA* mutant. The data show that anaerobic, fermentative growth of mutants in the AckA-Pta pathway is reduced but still possible. Growth reduction can be explained by the lack of an important ATP generating pathway of mixed acid fermentation. An *ackA* deletion mutant is more severely impaired than *pta* or *ackA-pta* deletion mutants. This is most probably due to the production of acetyl-P in the *ackA* mutant, leading to increased protein acetylation.

## Introduction

**FIGURE 1:**
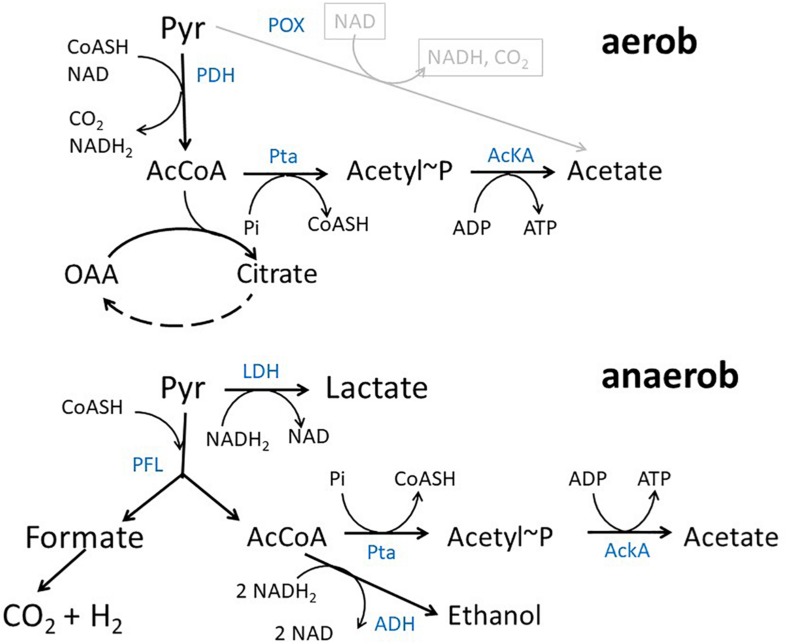
Acetate pathway in *E. coli.* Shown are the reactions connecting pyruvate, acetyl-CoA and acetate during growth with glucose under aerobic and anaerobic conditions, respectively. Blue text denotes enzymes PDH, pyruvate dehydrogenase complex; AckA, acetate kinase; Pta, phosphotransacetylase; LDH, lactate dehydrogenase; PFL, pyruvate-formate lyase; ADH, alcohol dehydrogenase; POX, Pyruvate Oxidase.

In *Escherichia coli* K12 acetate is a major product of metabolism under both aerobic and anaerobic growth conditions (Figure 1) (Wolfe, 2005; Bernal et al., 2016). Under aerobic conditions at high growth rates, e.g., during batch growth with glucose, acetate is produced by the so-called overflow metabolism. In exponential growth phase, acetate is predominantly produced by a pathway originating from acetyl-coenzyme A (acetyl-CoA) that is catalyzed by the enzymes phosphate acetyltransferase (encoded by *pta*) and acetate kinase (encoded by *ackA*) (Brown et al., 1977). The genes encoding the two proteins form an operon (Kakuda et al., 1994). The pathway produces acetate and ATP from acetyl-CoA, ADP and inorganic phosphate. There are a lot of ideas concerning the origin of aerobic acetate production, e.g., limitation of tricarboxylic acid cycle (TCA) capacity, limiting coenzyme A concentrations, limiting respiratory chain capacity or membrane space and proteome allocation (Majewski and Domach, 1990; Han et al., 1992; Varma and Palsson, 1994; Paalme et al., 1997; Pfeiffer et al., 2001; Vemuri et al., 2006; Veit et al., 2007; Molenaar et al., 2009; Valgepea et al., 2010; Renilla et al., 2012; Zhuang et al., 2014; Basan et al., 2015; Peebo et al., 2015; Szenk et al., 2017). The same pathway can be used in reverse direction for assimilation of acetate at medium or high external concentrations (Brown et al., 1977). It has been argued that acetate excretion and uptake are active concomitantly in *E. coli* strains showing overflow metabolism (Renilla et al., 2012; Enjalbert et al., 2017; Pinhal et al., 2019). Under anaerobic conditions, acetate represents a major product of mixed acid fermentation (Sawers and Clark, 2004). Under fermentative conditions, pyruvate produced by the glycolytic reactions is converted into acetyl-CoA and formate by the enzyme pyruvate formate lyase (PFL) (Sawers and Clark, 2004). Acetyl-CoA produced by this reaction can be converted to either acetate by an ATP generating pathway or to ethanol by a reaction consuming NADH. ATP production by the AckA-Pta pathway is important for growth under fermentative conditions. Except by the *ackA*-*pta* pathway acetate can be produced by the pyruvate oxidase PoxB, that is mainly active in stationary phase (Dittrich et al., 2008; Martínez-Gómez et al., 2012). PoxB is a common target in metabolic engineering approaches, too.

Mutants in the AckA-Pta pathway have been intensively analyzed before (el-Mansi and Holms, 1989; Diaz-Ricci et al., 1991; Yang et al., 1999; Contiero et al., 2000; Dittrich et al., 2008; Enjalbert et al., 2017) (for reviews see Wolfe, 2005; De Mey et al., 2007; Bernal et al., 2016). Concerning overflow the data show that in defined media with glucose as carbon source elimination of the AckA-Pta pathway has no major impact on aerobic growth yield and growth rate (Kakuda et al., 1994; Castaño-Cerezo et al., 2009). On the other hand, mutation of the AckA-Pta pathway has strong impact on anaerobic growth, strongly reducing the growth rate of the respective strains (Guest, 1979; Yun et al., 2005; Eydallin et al., 2014). Production of acetate and ATP from acetyl-CoA is blocked in both, the *ackA* and the *pta* mutants, but the mutants differ in the production of the intermediate acetyl-phosphate (acetyl-P). While *ackA* mutants can still produce this metabolite, *pta* mutants can’t. *ackA* mutants accumulate acetyl-P (McCleary and Stock, 1994; Prüß and Wolfe, 1994; Klein et al., 2007; Weinert et al., 2013). Acetyl-P has been related to global regulation because it can activate some response regulators of two-component systems, e.g., NRI, PhoB, and CheY (Feng et al., 1992; Wanner, 1992; McCleary et al., 1993; Wolfe, 2005; Fredericks et al., 2006), and because it is the acetyl donor for non-enzymatic protein lysine acetylation (Weinert et al., 2013; Kuhn et al., 2014; Schilling et al., 2015, 2019). Although both effects of acetyl-P have been convincingly demonstrated, their impact on cellular physiology is not fully elucidated, so far. Protein acetylation in *E. coli* has been studied on a global scale and acetylation of central metabolic enzymes is currently discussed as a mechanism to control metabolic fluxes (Weinert et al., 2013; Kuhn et al., 2014; Carabetta and Cristea, 2017; Nakayasu et al., 2017; Brunk et al., 2018; Christensen et al., 2019; VanDrisse and Escalante-Semerena, 2019). Besides such global effects, for a number of proteins an impact of acetylation on activity was shown (Wolfe, 2016; Carabetta and Cristea, 2017).

Acetate production competes with the production of other mixed acid products, e.g., succinate and lactate and acetate metabolism is hence a common target in attempts to optimize their production (Thakker et al., 2012; Förster and Gescher, 2014). The acetate pathway is also a target in the optimization of the production of other compounds, e.g., itaconat (Harder et al., 2016) or butanol (Atsumi et al., 2008). Also, acetate is known to hinder the production of recombinant proteins (Eiteman and Altman, 2006; Terol et al., 2019). If deletion of the acetate pathway is desired, deletion of *ackA*, of *pta* or of both genes might be favorable for a particular product or process. To elucidate in more detail the differences between the mutants, we performed a detailed analysis of the differences in physiology of Δ*ackA*, Δ*pta* and Δ*ackA-pta* mutants. Special focus was on anaerobic, fermentative growth conditions because these conditions are favorable for the production of organic acids and several other products (Thakker et al., 2012; Förster and Gescher, 2014). Furthermore, there are only limited investigations for these conditions.

In the publication at hand, isogenic *ackA*, *pta*, and *ackA*-*pta* mutants derived from MG1655 were constructed and physiologically characterized under anaerobic as well as aerobic batch growth with glucose as sole carbon source. We investigated glucose consumption and the production of fermentation products, as well as expression of selected genes. Moreover, we tested protein lysine acetylation. The data confirm previously published data for aerobic growth that show only limited impact of the acetate pathway on growth rate and yield in chemically defined medium with glucose as sole carbon source. Under anaerobic conditions, deletion of the acetate pathway caused a strong reduction of growth rate. The acetate mutants differed also with respect to the pattern of fermentation products, glucose uptake rate and ATP content. The *ackA* mutant showed a lower growth rate and lower specific glucose uptake rates than the other mutants. It also displayed differences in the expression of genes related to TCA, respiration and of the *atoDAEB* operon. Notably, this mutant showed an increased amount of protein lysine acetylation under anaerobic growth conditions as was to be expected (Weinert et al., 2013; Kuhn et al., 2014). Mass spectrometry based analysis of protein acetylation showed that increased protein lysine acetylation was especially pronounced for enzymes of glycolysis but also for enzymes like aspartate carbamoyl transferase, methionine synthase and catalase. For the last enzymes we could demonstrate that acetylation effected enzyme activity while we did not observe changes in the activities of the glycolytic enzymes GapA and FbaA. The data demonstrate that different mutations in the acetate pathway have an impact on physiology, especially under anaerobic conditions.

## Materials and Methods

### Strains and Culture Conditions

All strains are derivatives of *E. coli* K12 MG1655 and are listed in Table 1. Mutant strains were constructed using the method of Datsenko and Wanner (Datsenko and Wanner, 2000). Primers used for the knock-outs are listed in Supplementary Table 1. Resistance genes were eliminated as described (Datsenko and Wanner, 2000).

**TABLE 1:** Strains used in this work.

**Strain**	**Relevant Genotype**
MG1655	WT
KBM1081	MG1655 Δ*ackA*
KBM1082	MG1655 Δ*pta*
KBM1084	MG1655 Δ(*ackA-pta)*
KBM16121	MG1655 Δ*ackA* Δ*poxB*

For growth assays, cells were streaked on LB agar plates (10 g/l tryptone, 5 g/l yeast extract, 5 g/l NaCl and 15 g/l agar). A single colony was inoculated into 5 ml LB and incubated for about 4 h at 37°C with shaking. This culture was used to inoculate a shake flask with chemically defined medium [MM; 34 mM NaH_2_PO_4_, 64 mM K_2_HPO_4_, 20 mM (NH_4_)_2_SO_4_, 1 μM Fe(SO_4_)_4_, 300 μM MgSO_4_, 1 μM ZnCl_2_, 10 μM CaCl_2_] containing 4 or 2 g/l glucose (Tanaka et al., 1967) in a 1:100 dilution. If indicated, methionine was added to 40 μg/ml, serine to 20 mg/ml and uracil to 30 μg/ml. The shake flasks were incubated overnight at 37°C with shaking (for aerobic growth experiments) or without (for anaerobic growth experiments). These precultures were washed and transferred into fresh MM, again containing 4 g/l glucose. Main cultures were incubated at 37°C in shake flasks with vigorous shaking for aerobic experiments or in sealed bottles that were coupled to a N_2_ containing balloon and stirred slowly for anaerobic experiments.

For complementation of the mutant strains the sequence encoding *ackA-pta* was PCR amplified from the genome of MG1655 using Primers ackA-for-*Nde*I and pta-rev-*Hin*dIII (Supplementary Table 1) and Q5 polymerase (New England Biolabs). The PCR product was cloned into plasmid pRR48c (Studdert and Parkinson, 2005) using restriction enzymes *Nde*I and *Hin*dIII, resulting in pRR-ackA-pta. *pta* was deleted from this construct using the Q5 mutagenesis kit (New England Biolabs) and mutagenesis primers pRR-ackA-pta-mut-1 and 2 (Supplementary Table 1), resulting in pRR-ackA. Both constructs were verified by sequencing (Microsynth Seqlab, Göttingen, Germany).

### Measurements of Biomass and Extracellular Metabolites

Biomass was determined by measuring the OD_420_ of the cultures. An OD_420_ of 1 correlates to about 5^∗^10^8^ cells/ml and to 0.21 g/l dry cell weight. For determination of extracellular metabolites, samples were drawn, cooled and quickly centrifuged at 4°C for 2 min. Supernatants were stored at −20°C for further analysis. Glucose, ethanol, acetate, lactate and D-3-hydroxybutyric acid were determined enzymatically by using the respective test kits of Megazyme. A portion of the supernatants was filtered and analyzed by HPLC using Agilent 1100 Series with DAD detector (Agilent Technologies) with an Inertsil ODS-3 column (Gil Science Inc.) applying 0.1 M NH_4_H_2_PO_4_, pH 2.6 as solvent at a flow rate of 1 ml min^–1^. This chromatography allows for the quantification of formate, acetate, lactate, fumarate, and succinate as well as orotate.

### Measurement of Intracellular ATP

For determination of intracellular ATP concentrations 100 μl of cell culture were quickly transferred into boiling water and incubated for 10 min at 99°C with constant shaking at 600 rpm. Cell debris was removed by centrifugation for 10 min at 13000 rpm. ATP concentrations in the supernatant were analyzed by using the Celltiter-Glo 2.0 Assay of Promega according to the instructions of the manufacturer.

### Gene Expression Analysis by RT-qPCR

About 1.5 ^∗^10^9^ cells from exponential growth phase were quenched in twice the volume of RNAprotect Bacterial Reagent (Qiagen), vortexed for 5 s and incubated at room temperature for 5 min. Cells were pelleted by centrifugation, the supernatant was discarded and the pellet was stored at −80°C. RNA was prepared using the Master Pure RNA Purification Kit (Epicentre). RNA concentration and purity was determined using the NanoDrop spectrophotometer (Thermo Fisher Scientific).

mRNA was transcribed into cDNA by using the RevertAid H Minus First Strand cDNA synthesis Kit (Thermo Fisher Scientific). Quantitative PCR of different cDNA samples was performed using the MesaGreen qPCR Master Mix Plus (Eurogenetec) with SYBR Green as detection agent and the Rotor-Gene 6000 (Corbett Life Science). Primer sequences used are listed in Supplementary Table 1. Amplification conditions were: 95°C for 10 min, followed by 40 cycles at 95°C for 15 s and 60°C for 1 min. A negative control without template was conducted for each primer pair in each PCR run and a control for DNA contamination was performed for each RNA sample used. Quantification was performed by relative quantification to housekeeping genes (*rpoD*, *yhbc*, and *recA*) applying the ΔΔCt method (Livak and Schmittgen, 2001; Hellemans et al., 2007) with efficiency correction.

### Determination of Protein Lysine Acetylation by Western Blotting

About 5^∗^10^8^ cells were harvested from mid exponential phase and extracted with BugBuster protein extraction reagent (Millipore) for 20 min at room temperature. 100 μL of SDS sample buffer (1% SDS, 2.5% 2-mercaptoethanol, 31.25 mM Tris-HCl pH 8.0, 5% glycerol, 0.0025% bromophenolblue) was added. 15 μl of extract was separated by SDS-PAGE using 12% polyacrylamide gels (Laemmli, 1970). Proteins were transferred to a Nylon membrane (0.2 μm, Millipore) by semi-dry blotting at 100 mA per gel for 20 min using transfer buffer (20% methanol, 1.3 mM SDS, 192 mM glycerol, 25 mM Tris). To check for equal protein amounts in the extracts, 5 μl of each extract was seperated by SDS-PAGE under the same conditions as above but stained with Coomassie brilliant blue.

Membranes were blocked with PBS 0.1% Tween 20 with 3% low fat milk powder in and probed with Acetylated Lysine Multi MAB (Cell Signaling Technologies) again in PBS 0.1% Tween with 3% low fat milk powder. As secondary antibody HRP labeled goat anti-rabbit secondary antibody (Sigma) was used in PBS 0.1% Tween 20. Signals were detected by using Super Signal West Femto Maximum Sensitivity Substrate (Thermo Fisher Scientific) and a CCD camera (Intas).

### Cell Lysis and Protein Determination

To compare protein abundance and acetylation in KBM1081 with MG1655, cells were grown under anaerobic conditions as described above. At mid-log phase cells were harvested, washed and resuspended in TE buffer (10 mM Tris, 1 mM EDTA, pH 7.5). Cells were lysed by sonification (BRANSON Digital Sonifier). Extracts were clarified by centrifugation at 13000 rpm for 30 min and protein concentrations were determined according to Bradford (Bradford, 1976).

### Filter-Aided Sample Preparation (FASP) Digestion

For FASP digestion (Wiśniewski et al., 2009), protein extract containing 100 μg protein was loaded onto the FASP filter (Pall Nanosep 10K Omega, MWCO 10 kDa) and centrifuged [10–20 min, room temperature (RT), 10000 *g*]. Reduction with dithiothreitol (20 min, 56°C, 300 rpm) and alkylation with iodoacetamide (20 min, RT, 300 rpm, in the dark) of proteins was carried out by addition of 100 μL 40 mM dithiothreitol in urea buffer (8 M urea in 0.1 M Tris-HCl pH 8.5) and 100 μL 55 mM iodoacetamide in urea buffer. After each of these steps the liquid was removed by centrifugation (5 min, RT, 10000 *g*) and the flow through was discarded. Subsequently, the proteins were washed for 2 min with 100 μL urea buffer, three times with 100 μL 50 mM ammonium bicarbonate, and centrifuged afterward (5 min, RT, 10000 *g*). After removal of the flow through, 200 μl trypsin buffer (50 mM ammonium bicarbonate, enzyme to protein ratio of approximately 1–100) was added onto the FASP filter (2 h, 37°C, 300 rpm). Subsequently, the sample was centrifuged (5 min, RT, 10000 g). Remaining peptides were rinsed through the filter by addition of 50 μL 50 mM ammonium bicarbonate and 50 μL ultrapure water (Millipore Q-POD Merck, Darmstadt, Germany) followed by another centrifugation step (5 min, RT, 10,000 *g*). Finally, 30 μL were acidified by addition of 3 μL 0.5% trifluoroacetic acid (TFA), centrifuged (10 min, 4°C, 10000 *g*), and transferred into an HPLC vial.

### Liquid Chromatography

One μL of each sample was injected and separated by UltiMate^®^ 3000 nano splitless reversed phase nanoHPLC (Thermo Fisher Scientific, Dreieich) equipped with a reversed phase trap column (nano trap cartridge, 300 μm i.d. ×5 mm, packed with Acclaim PepMap100 C18, 5 μm, 100 Å, nanoViper, Bremen, Germany) and a reversed phase separation column (Acclaim PepMap RSLC, C18, 2 μm, 75 μm, 50 cm, Bremen, Germany). The gradient was 5–35% mobile phase B (acetonitrile, 0.1% formic acid) over 120 min at a flow rate of 0.4 μL min^–1^. The LC was coupled directly to the MS.

### Mass Spectrometry

The timsTOF^TM^ mass spectrometer (Bruker Daltonik GmbH, Bremen) was equipped with a captive spray ionization (CSI) source operated in positive ion mode with a capillary voltage of 1400 V, 3 l/min dry gas and 200°C dry temperature.

For each of the two sample types a data dependent MS/MS spectra acquisition (DDA) in TOF mode was performed with a spectra rate of 2 Hz in a cycle of 3 s over the mass range from 150 to 2200 m/z using collision induced dissociation (CID) with a smart exclusion value of 5 and an active exclusion of the same precursor after 1 spectra for 30 s or a release if intensity/previous intensity exceeded 3. Precursor with charge state 2–5 were recorded, singly or unknown charged precursors were excluded.

For data independent (DIA) SWATH-MS (sequential window acquisition of all theoretical spectra) experiments, the acquisition was performed in TOF mode covering the mass range from 400 to 1408 m/z with 32 precursor isolation windows with 25 m/z width for 400–1000 m/z and 51 m/z for 1000–1408 m/z (1 m/z overlap for each window) with a spectra rate of 10 Hz, resulting in 3.2 s cycling time. The collision energy was stepwise increased for the distinct precursor isolation windows from 7 to 48 eV.

### Data Analysis

Results of the DDA experiments were processed as ^∗^mgf files followed by a MASCOT search^1^ against the reviewed Swiss-Prot protein database for *E. coli* K12 strain (2018-07-02, 4475 protein entries), using carbamidomethylation of cysteine residues as fixed, as well as acetylation (Lys, N-term) and oxidation of methionine as variable modification. Further preferences were: enzyme: trypsin, missed cleavage: 1, peptide charge: +2, +3, +4, peptide tolerance: 0.02 Da, MS/MS tolerance 0.2 Da, #13C: 0.

Data analysis was performed using Skyline 4.2 (MacCoss Lab, University of Washington) (MacLean et al., 2010). A spectral library was set up using MASCOT search results (^∗^.dat files) of the two DDA measurements and importing of the *E. coli* K12 Swiss-Prot database as a background proteome. For importing Bruker ^∗^.raw files of the SWATH-MS measurements the isolation scheme in Skyline was set as the SWATH precursor isolation windows used in the acquisition. Only scans within 5 min of MS/MS IDs were considered and MS/MS ions were filtered for the 5 most intense y-ions. Further preferences were: mass accuracy: 30 ppm, product mass analyzer: centroided, enzyme: trypsin, missed cleavages: 1, peptide length: 6–45, structural modifications: carbamidomethyl (Cys), oxidation (Met), acetyl (Lys, N-term), carboxymethyl (N-term).

Peptide data was exported to Microsoft Excel (version 2010) for final data analysis. Peptides with signal intensities less 50.000 (noise) were deleted to focus on valuable data. Proteins were quantified by summing up the peptide intensities (peak areas) of corresponding peptides. In addition, acetylated peptides were considered separately. Acetylation ratio of proteins was calculated in percent of total intensities of the proteins. Protein abundances and acetylation ratios were compared between both sample groups by calculating quotients.

### Enzyme Activity Assays

Pyruvate formate lyase activity was determined essentially as described by Gupta and Clark (1989). Cells were grown in an anaerobic jar on LB plates containing 0.2% glucose overnight at 37°C. Cells were overlaid with soft agar (0.75%) containing 25 mM potassium phosphate buffer (pH 7.4), 100 mM pyruvate and 1 mg/ml benzylviologen. After about 5 min the color was checked.

Catalase was assayed by a visual approach as described by Iwase et al. (2013). For each repetition the activity of MG1655 was set to 100%.

## Results

### Anaerobic Growth Characteristics of *ackA, pta, and ackA-pta*- Mutants

**FIGURE 2:**
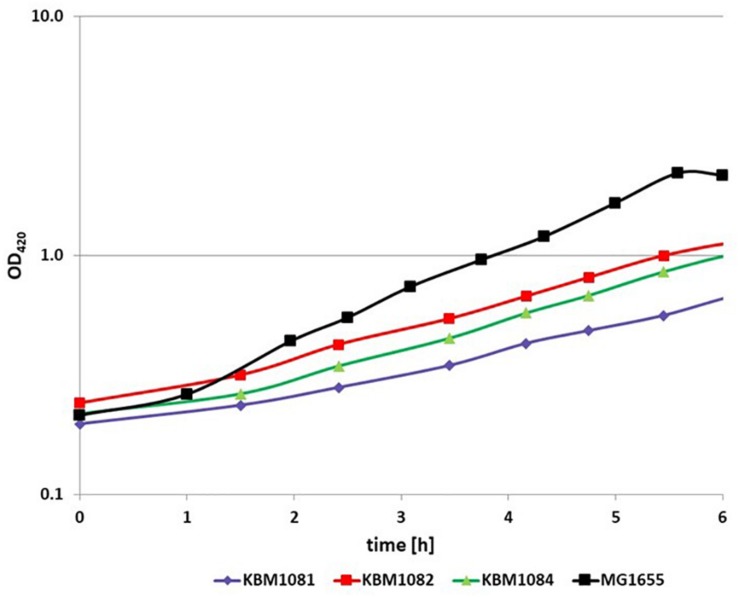
Anaerobic growth of MG1655, KBM1081, KBM1082, and KBM1084. Cells were grown anaerobically in defined medium with 4 g/l glucose. Shown are exemplary time course data for the OD_420_ from batch cultures incubated under N_2_ atmosphere. The growth assays were performed in at least three independent experiments. Variation of these repeats and further data concerning substrate consumption and production of fermentation products can be seen from Table 2.

To analyze the fermentative growth of *ackA*, *pta*, and *ackA-pta* deletion mutants, we investigated growth of isogenic mutants (Table 1) in defined medium with glucose as sole carbon source All three mutants showed significantly reduced growth rates and a lower biomass yield (Figure 2 and Table 2). KBM1081, the *ackA* mutant, displayed the slowest growth rate of all strains tested. This mutant also showed a lower glucose uptake rate than the other strains (8.7 mmol per gram cell weight compared to about 17 mmol per gram cell weight and hour) (Table 2). While acetate production is almost completely absent in the *pta* and the *ackA-pta* mutant, the *ackA* mutant still showed residual acetate production. Acetate can also be produced by the pyruvate:ubiquinone oxidoreductase, PoxB (Figure 1). To check if PoxB is involved in acetate production in the *ackA* mutant, we constructed the *poxB ackA* deletion mutant KBM16121 (Table 1). Adding the *poxB* deletion did not reduce acetate production (Table 2).

**TABLE 2:** Growth characteristics of MG1655 and the acetate mutants under anaerobic conditions.

	**μ**	**Y_bio_**	**Y_ace_**	**Y_lact_**	**Y_suc_**	**Y_EtOH_**	**Y_Form_**	**Glc_up**
								
	**h^–1^**	**g/g**	**mol/mol_Glc_**	**mol/mol_Glc_**	**mol/mol_Glc_**	**mol/mol_Glc_**	**mol/mol_Glc_**	**mmol/(g^∗^h)**
MG1655	0.43 ± 0.01	0.14 ± 0.02	0.69 ± 0.04	0.02 ± 0,003	0.15 ± 0.04	0.59 ± 0.04	1.03 ± 0.06	17.8 ± 3.5
KBM1081	0.17 ± 0.03	0.09 ± 0.01	0.25 ± 0.08	1.12 ± 0.04	0.15 ± 0.02	0.18 ± 0.01	0.28 ± 0.08	8.74 ± 0.4
KBM1082	0.27 ± 0.03	0.09 ± 0.02	0.02 ± 0.00	1,35 ± 0.1	0.10 ± 0.02	0.12 ± 0.04	0.11 ± 0.02	17.8 ± 2.2
KBM1084	0.28 ± 0.01	0.08 ± 0.01	0.03 ± 0.01	1.38 ± 0.11	0.10 ± 0.02	0.12 ± 0.03	0.10 ± 0.04	18.2 ± 0.9
KBM16121	0.16 ± 0.01	0.08 ± 0.01	0.25 ± 0.06	0.93 ± 0.01	0.14 ± 0.03	0.1*	0.3 ± 0.02	11.1 ± 0.3

Data are derived from at least three independent growth curves with exception of data for KBM16121, which were determined from only two independent experiments. *This value was only measured once.

All three acetate mutants produced lactate as the major fermentation product, which was produced only in low amounts by MG1655 (Table 2). The amounts of the other fermentation products changed, too. The production of ethanol and formate was reduced compared to MG1655. Acetate, formate, ethanol and succinate production was higher in the *ackA* mutant KBM1081, than in the *pta* mutant KBM1082 and the *ackA*-*pta* mutant, KBM1084.

As the acetate pathway is an important ATP generating pathway under anaerobic conditions, we determined ATP concentrations in the cells. The measured ATP concentrations reflected the growth rate of the cells. For MG1655 an ATP concentration of 5.2 ± 0.16 μmol/gDCW was determined. The *ackA-pta* mutant KBM1084 showed a concentration of 4.2 ± 0.7 μmol/gDCW and the *ackA* mutant KBM1081 a clearly lower concentration of 2.8 ± 0.8 μmol/gDCW.

To verify that the phenotypes observed were solely due to the *ackA* and *pta* mutations, the mutants were complemented with pRR-ackA-pta or pRR-ackA. These are low copy plasmids encoding *ackA-pta* or only *ackA*, respectively. Introduction of the plasmids restored growth and acetate production in all mutants (Supplementary Table 2).

### Aerobic Growth of *ackA*, *pta*, and *ackA-pta*- Mutants

For the sake of completeness, we analyzed the *ackA, pta* and *ackA-pta* deletion mutants (Table 1) also for aerobic batch growth with glucose. The mutants impaired in acetate overflow grew slightly slower on glucose than MG1655 (Table 3). This was accompanied by slightly reduced glucose uptake rates in the mutant strains. Again, both effects were more pronounced for the *ackA* mutant KBM1081. The difference in growth rate between the three mutants cannot be due to impairment of acetate overflow in general, as overflow is eliminated in all mutants alike. Slow growth of KBM1081 might therefore be explained with elevated intracellular acetyl-P levels. Biomass yield for all three mutants was comparable to the wild type, even though MG1655 produced significant amounts of acetate.

**TABLE 3:** Growth characteristics of MG1655 and the acetate mutants under aerobic conditions.

	**μ**	**Y_bio_**	**Y_ace_**	**Glc_up**
				
	**h^–1^**	**g/g**	**mol/mol_Glc_**	**mmol/(g^∗^h)**
MG1655	0.75 ± 0.01	0.41 ± 0.02	0.34 ± 0.014	10.5 ± 0.3
KBM1081	0.63 ± 0.06	0.41 ± 0.01	0.08 ± 0.03	8.4 ± 0.6
KBM1082	0.68 ± 0.06	0.40 ± 0.06	0.06 ± 0.00	9.3 ± 0.7
KBM1084	0.68 ± 0.07	0.40 ± 0.03	0.06 ± 0.01	9.6 ± 0.8

### Analysis of Gene Expression and Protein Amounts

Since under anaerobic conditions clear differences between MG1655 and the mutant strains became obvious, we analyzed the expression of selected genes by qRT-PCR and compared protein amounts in MG1655 and in the *ackA* mutant KBM1081 via mass spectrometry.

**FIGURE 3:**
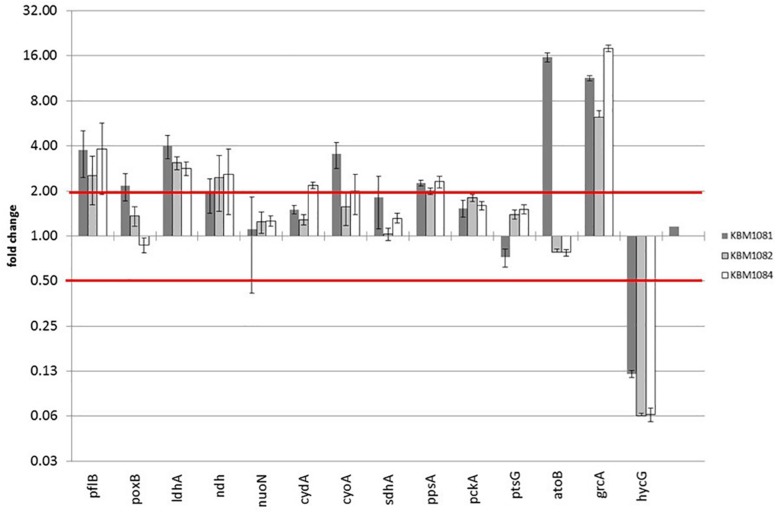
Gene expression data of acetate mutants during anaerobic growth with glucose. Strains were grown anaerobically in MM with 4 g/l glucose. RNA was extracted during exponential growth phase and gene expression was analyzed by qRT-PCR as described. Relative changes in gene expression were calculated by the ΔΔCt method using MG1655 grown under the same conditions as reference sample. Three housekeeping genes *rpoD*, *yhbc* and *recA* were used for normalization. Shown are only selected genes.

On the protein level in total 31 proteins were expressed at least twofold higher in KBM1081 than in MG1655 (Supplementary Table 3) and 33 proteins were expressed at least two-fold lower in KBM1081 than in MG1655 (Supplementary Table 4). Notably, three proteins of the *atoDAEB* operon for acetoacetate degradation (Pauli and Overath, 1972) showed strong differences in the two strains. Analysis of *atoB* expression via RT-PCR confirmed the proteomic results (Figure 3 and Supplementary Table 5). *atoB* expression was significantly upregulated in KBM1081 but not in the other mutants. As the *ato* operon is involved in acetoacetate as well as in 3-hydroxybutyric acid (3HB) synthesis, both metabolites were analyzed in supernatants from anaerobic growth curves with KBM1081 (*ackA*), KBM1084 (*ackA pta*) and MG1655 (wt). Acetoacetate levels were below detection limit in all strains (data not shown). However, low but increased production of 3HB was detected for the *ackA* mutant (Supplementary Figure 1). The proteome data also showed an increased amount of GrcA, a stress induced alternate pyruvate-formate lyase subunit (Wyborn et al., 2002). The qRT-PCR data could confirm an increased expression of *grcA* in all mutant strains (Figure 3 and Supplementary Table 5). The amount and expression of PflB was increased in the mutants, too. HycG, a subunit of formate hydrogen lyase was strongly downregulated in all mutants (Figure 3 and Supplementary Tables 3, 5). This might be explained by the reduced production of formate in the mutants, rendering formate removal unnecessary (Rossmann et al., 1991). Matching the increased production of lactate, *ldhA* expression was increased in the mutants.

For qRT-PCR we additionally chose genes associated with acetate metabolism, fermentation, TCA, anaplerosis, respiratory chain as well as some genes for regulatory proteins. Data for all genes tested is displayed in Supplementary Table 5. A slight increase in *poxB* (encoding pyruvate oxidase) expression was observed in the *ackA* mutant KBM1081 (Figure 3). Gene *ndh*, encoding NADH dehydrogenase II, was slightly upregulated in all mutant strains, while other genes involved in respiratory chain or TCA like *nuoN*, *icdA*, *cydA*, and *sucA* did not change significantly (Figure 3). In KBM1081 upregulation of *cyoA* (encoding terminal oxidase bo) was observed by qPCR (Figure 3). In all three mutants *ppsA*, the gene encoding the gluconeogenic PEP synthase, was slightly upregulated (Figure 3). This most probably reflects an accumulation of pyruvate in the cells, which is also reflected by the production of lactate. It is doubtful, if PEP synthase is actually active in the mutants.

A small difference in gene expression was also observed for *ptsG*, encoding IICB^Glc^, the membrane component of the glucose-PTS. The slight reduction in *ptsG* expression fits to the reduced glucose uptake in this mutant.

### Analysis of Protein Lysine Acetylation

**FIGURE 4:**
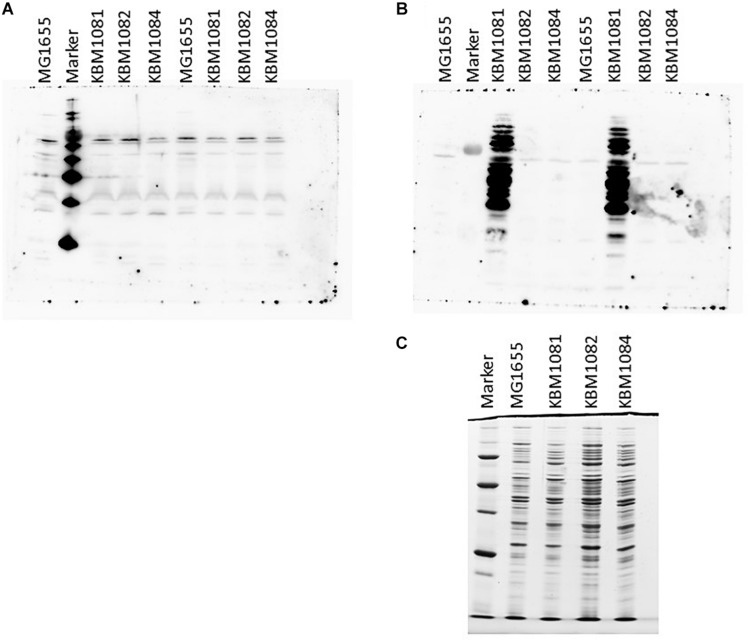
Protein lysine acetylation in MG1655, KBM1081, KBM1082, and KBM1084. Shown are Western Blots of gels probed with Acetylated Lysine Multi MAB with extracts from exponentially growing aerobic cultures **(A)** and anaerobic cultures **(B)**. **(C)** Shows the Coomassie stained loading control for the anaerobic extracts used in **(B)**. For the Western Blots shown in **(A,B)** 15 μl of the respective extracts were loaded per lane. For the loading control in **(C)** only 5 μl of the extracts were loaded. As markers MagicMark XP Western Protein Standard (Thermo Fisher Scientific) was used **(A)** and Precision Plus Unstained Protein (BioRad) for **(B,C)**. Culture growth and extract preparation were performed as described in Section “Materials and Methods.”

As *ackA* mutants have been shown to manifest increased protein lysine acetylation under aerobic conditions probably due to the accumulation of acetyl-P (Weinert et al., 2013; Kuhn et al., 2014; Schilling et al., 2015), we checked protein lysine acetylation in the wild type and the mutant strains. To this end, we harvested cells actively growing under either aerobic or anaerobic conditions in mid-exponential phase and the proteins were separated by SDS-PAGE. After Western blotting, the membranes were probed with antibodies against acetylated lysine. As can be seen in Figure 4A, only few bands were detected for aerobic growth and no obvious differences between the wild type and the mutants were observable. This fits nicely to the data of Schilling et al. (2015), who observed significant amounts of lysine acetylation in stationary cells only.

A different observation was made under anaerobic conditions. While MG1655, KBM1082 (*pta*) and KBM1084 (*ackA pta*) again showed a low level of protein lysine acetylation, acetylation strongly increased in KBM1081 (*ackA*) (Figure 4B). Acetyl-P is known to act as an acetyl donor for protein acetylation (Weinert et al., 2013; Kuhn et al., 2014). An increased intracellular acetyl-P level, as expected for the *ackA* mutant KBM1081 (Weinert et al., 2013), might hence result in increased protein acetylation. In KBM1081 growing anaerobically, significant protein acetylation can be observed during the active growth phase of the strain. The results indicate either a stronger accumulation of acetyl-P in KBM1081 during anaerobic growth than during aerobic growth or protein lysine acetylation might be increased due to other mechanisms in this mutant under anaerobic conditions.

To analyze protein lysine acetylation in more detail, protein extracts were prepared from anaerobically growing cultures of MG1655 and KBM1081. The extracts were tryptically digested and submitted to nanoHPLC-MS/MS in TOF mode and SWATH mode. This method allows the relative quantification of proteins in both samples, as well as the determination of the acetylation sites of specific peptides. Peptides displaying significant acetylation in one of the samples are listed in Supplementary Table Acetylated Peptides. Some proteins showed a higher acetylation in KBM1081 than in MG1655 (Table 4). Several glycolytic enzymes, e.g., enolase, glyceraldehyde 3-P dehydrogenase, fructose-bisphosphate aldolase, pyruvate-formate lyase and phosphoglycerate kinase showed high differential acetylation.

**TABLE 4:** Proteins showing differential acetylation in KBM1081 vs. MG1655.

**Protein**	**Acetylated Peptides**	**Ratio KBM1081:MG1655**
Eno	9	44.5/16.0/13.7/9.5/8.6/4.1/2.9/1.7/1.3
KatE	1	43.1
GapA	6	17.1/13.3/12.1/6.5/1.8/1.7
Tsf (EF-Ts)	1	17
TufA (EF-Tu)	5	12.4/5.6/5.4/2.4/0.9
FbaA	1	10.6
PflB	4	10.2/8.9/5.6/2.5
Pgk	5	9.6/1.7/1.3/0.24/0.2
PyrB	2	9.1/7.2
GatY	1	8.2
PtsI	1	7.5
AdhE	1	6.5
WrbA	1	4.8
DapD	1	4.5
RplI	1	4.4
GroL	2	4.2/0.9
SerA	1	4.1
MetE	2	3.5/0.3
AtpA	1	3.1
LysS	1	3.0
PykF	3	2.8/1.4/1.1
Upp	1	2.7
GadA	2	1.1/2.6
Crr	1	2.5
FbaB	1	2.4
GlyA	1	2.1
PtsH	1	2
ElaB	1	2
InsJ	1	0.5
PurK	1	0.5
KatG	1	0.4
YceF	1	0.4
LuxS	1	0.3
RplL	1	0.1
Dps	1	0.1

*Listed are proteins containing at least one peptide with a twofold difference in acetylation level between both strains. Proteins are sorted according to the highest differences in peptide acetylation.*

To check if acetylation influences enzyme activities, we analyzed activities of glyceraldehyde 3-phosphate dehydrogenase and fructose-bisphosphate aldolase in extracts from anaerobically grown MG1655 and KBM1081 but the activity of both enzymes was the same in both strains (data not shown). We also analyzed pyruvate formate lyase activity by a qualitative plate assay (Gupta and Clark, 1989). The results indicated reduced but measurable PFL activity in KBM1081 but no activity in KBM1084 (Supplementary Figure 2). This reflects the low production of acetate, formate and ethanol in KBM1081 compared to undetectable production in KBM1084.

The proteins of the phosphotransferase system involved in glucose uptake, enzyme I, EIIA^Glc^ and HPr, showed significantly higher acetylation in KBM1081 than in MG1655.

**FIGURE 5:**
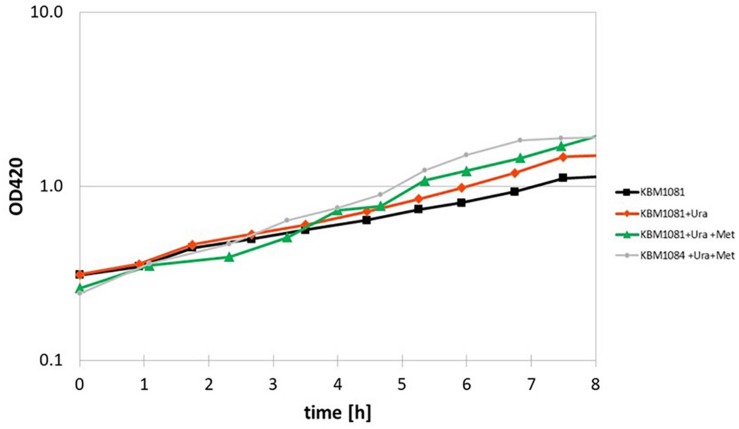
Anaerobic growth of the *ackA* mutant KBM1081 in defined medium supplemented with uracil and methionine. Shown are time course data from representative growth assays for growth of the *ackA* mutant KBM1081 in defined medium with 4 g/l glucose as carbon source without any further growth supplements (black) and with addition of 30 μg/ml uracil (red) and with addition of 30 μg/ml uracil plus 40 mg/l L-methionine (green). Growth of the *ackA*-*pta* mutant KBM1084 with supplementation of uracil and methionine is shown for comparison (gray).

Another metabolic enzyme showing strong differences in acetylation is aspartate carbamoyl transferase (PyrB), an enzyme involved in uracil (pyrimidine) biosynthesis. An inhibition of this enzyme by acetylation could well explain the slow growth of KBM1081. Motivated by this result, we analyzed growth of KBM1081 and KBM1084 in MM glucose with and without addition of uracil. While addition of uracil to the growth assays had no influence on the growth rate of KBM1084, the growth rate of KBM1081 increased after addition of uracil (from 0.17 h^–1^ to 0.2 h^–1^) (Figure 5 and Supplementary Figure 3).

A differential acetylation was also observed for the cobalamin-independent homocysteine transmethylase (methionine synthase, MetE), an essential enzyme in methionine biosynthesis. An inhibition of MetE by acetylation could contribute to the low growth rate of the *ackA* mutant. Supplemented with uracil and methionine, the *ackA* mutant reached a growth rate of 0.249 ± 0.02 h^–1^, thereby reaching a similar growth rate than the *pta* mutant (Figure 5). Supplementation of the Δ*ackA-pta* mutant KBM1084 with uracil and methionine did not increase growth rate (Supplementary Figure 3).

Differential acetylation was also observed for SerA, catalyzing the first step in L-serine biosynthesis. The addition of L-serine, however, did not improve growth of the *ackA* mutant and the growth rate remained at 0.17 h^–1^ (data not shown).

Besides the metabolic enzymes mentioned, also a number of proteins involved in translation and stress response showed higher acetylation. Catalase HPII (KatE) showed strong acetylation. We hence tested catalase activity by a qualitative assay (Iwase et al., 2013). Compared to MG1655 (100%), catalase activity in KBM1081 increased to 168.5% (±20.5%), while catalase activity in KBM1084 was comparable to MG1655 (99.2% ± 5.5%).

## Discussion

The acetate pathway is a common target in metabolic engineering approaches as acetate production competes with the production of other organic acids and several products of biotechnological interest (Atsumi et al., 2008; Thakker et al., 2012; Förster and Gescher, 2014; Harder et al., 2016). This study presents a systematic comparison of isogenic mutant strains growing on glucose in defined medium with focus on anaerobic conditions. The data show significant differences between the different mutants.

The reasons for acetate overflow have been investigated in several studies and different explanations were discussed (Majewski and Domach, 1990; Varma and Palsson, 1994; Pfeiffer et al., 2001; El-Mansi, 2005; Wolfe, 2005; Vemuri et al., 2006; Molenaar et al., 2009; Valgepea et al., 2010; Renilla et al., 2012; Zhuang et al., 2014; Basan et al., 2015; Peebo et al., 2015). Our results show that in aerobic batch growth with glucose prevention of aerobic overflow reduces the growth rate of MG1655 by 10–15%. It has been reported that the relation between acetate production and respiration adjusts as a consequence of proteome allocation (Basan et al., 2015). The 10% higher growth rates achieved by the wild type as compared to the mutants should hence reflect the advantage of cooperation between respiration and substrate level phosphorylation. An alternative explanation might be the accumulation of acetyl-CoA, slowing down growth of the acetate mutants due to a drop in the free CoA pool. A low CoA pool limits the activity of the 2-ketoglutarate dehydrogenase complex and reduces the flux through the TCA. Limitation of CoA supply has been discussed as a reason for overflow metabolism, too (El-Mansi, 2004, 2005). Different to previous studies with BL21 (Castaño-Cerezo et al., 2009), in our hands the mutants did not show an enhanced lactate production.

Differences between the mutants and the wild type became more visible under anaerobic conditions. The reduced biomass yield of all mutants might be explained by the omission of an important ATP production pathway. In addition to acetate production, ethanol and formate production was strongly reduced in the mutant strains. Acetate and ethanol are both derived from acetyl-CoA. Anaerobically, acetyl-CoA and formate are the products of the PFL reaction (Figure 1). Due to the missing acetate pathway, acetyl-CoA might accumulate in the cells and thereby inhibit the PFL reaction. The reduced PFL activity is in contrast to increased levels of PFL in the *ackA* mutant (Supplementary Table 3) and to the increased transcription of *pflB* in all mutant strains (Figure 3 and Supplementary Table 5). An inhibition of the PFL reaction by accumulating acetyl-CoA, might in turn lead to an accumulation of pyruvate (Figure 1). Pyruvate is known to induce *pflB* expression (Sawers and Böck, 1988) and pyruvate accumulation might explain the increased expression and protein amounts in the mutant strains. This is also reflected by the PFL activity assay (Supplementary Figure 2) that showed no activity in the Δ*ackA-pta* mutant, KBM1084. Low PFL activity could be detected in KBM1081, in agreement with the observed higher production of acetate and formate detected for this mutant. This strain can convert part of the acetyl-CoA into the intermediate acetyl-P that might hydrolyze to acetate (Brown et al., 1977), thereby relieving in part the inhibition of PFL. Hydrolysis of acetyl-P is not coupled to ATP generation and might explain the low ATP content of the *ackA* mutant.

In agreement with the reduced formate production, expression of *hycG*, encoding a subunit of formate hydrogen lyase (Rossmann et al., 1991) is strongly reduced in the mutants (Figure 3 and Supplementary Table 5). As discussed above *pflB* transcription is increased in these strains. Also, expression of *grcA* (synonym *yfiD)*, encoding an alternative, stress induced PFL subunit (Wagner et al., 2001; Zhu et al., 2007), is strongly increased in the mutants. Expression of *yfiD* is influenced by many “stress” factors and might be the answer to the accumulation of pyruvate or other acidic products (Blankenhorn and Phillips, 1999; Wyborn et al., 2002).

The increased abundance of three proteins encoded by the *atoDEAB* operon is related to acetoacetate metabolism (Pauli and Overath, 1972; Jenkins and Nunn, 1987). As carbon source, acetoacetate is degraded into acetoacetyl-CoA, which is cleaved into two molecules of acetyl-CoA. This reaction is reversible. Accumulation of acetyl-CoA and acetyl-P in KBM1081 might promote the backwards reaction, namely the synthesis of acetaoacetyl-CoA and possibly its subsequent degradation to acetoacetate. The *atoDEAB* operon is under control of the AtoCS two component signal transduction system (Jenkins and Nunn, 1987). An internal induction of the *atoDEAB* operon in *ackA* mutants could explain the observed effects. The Ato enzymes are involved in 3HB production and small amounts of this component could be detected in supernatants of KBM1081 (Supplementary Figure 1). For biotechnological approaches that aim at production of 3HB or related compounds, *ackA* mutants might hence be preferred to other acetate mutants. The AtoCA system has been implicated in the control of flagella synthesis and motility (Oshima et al., 2002; Theodorou et al., 2012). The *ackA* mutation affects motility and biofilm formation (Prüß and Wolfe, 1994; Wolfe et al., 2003). The Rcs phosphorelay system has been shown to link acetyl-P with the synthesis of flagellar and capsular proteins but it was hypothesized that an additional acetyl-P sensitive regulator might be involved (Fredericks et al., 2006). The AtoCS system could be this additional link.

Accumulation of acetyl-P has been correlated with non-enzymatic protein lysine-acetylation (Weinert et al., 2013; Kuhn et al., 2014). Confirming previous studies (Schilling et al., 2015), protein acetylation was not increased during exponential aerobic growth whereas strong acetylation occurred in the *ackA* mutant during anaerobic growth. Most of the about 30 proteins showing increased acetylation in the *ackA* mutant have already been identified as being subject to lysine acetylation (Yu et al., 2008; Weinert et al., 2013; Zhang et al., 2013; Kuhn et al., 2014; Nakayasu et al., 2017; Brunk et al., 2018). Accordingly, proteins involved in glucose metabolism and uptake show increased acetylation in KBM1081 growing anaerobically (Table 4 and Supplementary Table Acetylated Peptides). Enolase, glyceraldehyde 3-P dehydrogenase, fructose-bisphosphate aldolase, phosphoglycerate kinase, pyruvate kinase from glycolysis, as well as Enzyme I, HPr and EIIA^Glc^ from the phosphotransferase system were detected in studies analyzing protein acetylation under aerobic conditions (Weinert et al., 2013; Kuhn et al., 2014; Schilling et al., 2015; Brunk et al., 2018). It is still unclear whether acetylation of central metabolic proteins represents a general of means of *E. coli* to fine tune glycolysis flux or if enzymes are targeted unspecifically (Weinert et al., 2013; Kuhn et al., 2014; Schilling et al., 2015, 2019; Nakayasu et al., 2017; Brunk et al., 2018). According to Kuhn et al. (2014) acetylation of K184 of GapA decreases the enzyme’s affinity for NAD. This acetylation site was detected in KBM1081, too. Acetylation of enolase that was also found in KBM1081, was reported to reduce its activity (Nakayasu et al., 2017; Brunk et al., 2018). Using standard assays, enzymatic activities of glyceraldehyde 3-P dehydrogenase and fructose-bisphosphate aldolase were similar in MG1655 and KBM1081. In experiments acetylation of proteins is often mimicked by mutation of a single amino acid (Kuhn et al., 2014; Brunk et al., 2018). While such mutations result in a stable change of all enzyme molecules, acetylation will most probably affect only a portion of the molecules. In addition, one cannot exclude, that such mutations have more severe impact on the protein activity than acetylation. Also, changed affinities for cofactors might not become visible in standard enzyme assays but might still reduce fluxes in central metabolism. The reduced glucose uptake rate as observed for the *ackA* mutant might, however, result from acetylation of PTS enzymes and glycolytic enzymes.

Aspartate carbamoyl transferase was identified as another protein showing strongly increased acetylation in the *ackA* mutant. A lysine residue in the active site was acetylated (Wang et al., 2005). This enzyme is part of the uracil biosynthesis pathway. Supplementation of uracil partly restored the anaerobic growth rate of the *ackA* mutant, KBM1081 (Figure 5 and Supplementary Figure 3), thereby further confirming an effect of acetylation on the activity of aspartate carbamoyl transferase. Another anabolic enzyme, the methionine synthase, MetE, was acetylated in the *ackA* mutant, too. Supplementation of methionine to minimal medium allowed for faster anaerobic growth of the *ackA* mutant. Supplementation of the medium with both, uracil and methionine, resulted in a growth rate of the *ackA* mutant similar to the growth rates of the *pta* and *ackA-pta* mutants (Figure 5). Acetylation of essential anabolic key enzymes hence was the reason for the slow growth phenotype of the *ackA* mutant KBM1081 in minimal medium under fermentative conditions.

The increased acetylation of catalase in KBM1081 and higher catalase activity in KBM1081 than in MG1655 or KBM1084 might be an answer to increased stress provoked by the slow growth or by methionine and uracil starvation.

Our data demonstrate that there are significant differences between an *ackA* mutant, compared to *pta* and *ackA-pta* mutants. With respect to strain design for biotechnological applications, the differences between the mutants might prove important. The differences between the mutants are most likely caused by an accumulation of acetyl-P in the *ackA* mutant. We could show that the slow growth of the *ackA* mutant is at least partly provoked by limitations in the methionine and uracil biosynthesis pathways. The question if protein lysine acetylation is a means to control metabolic fluxes still has to be resolved. Most probably, only few acetylation sites have an impact on growth and enzyme activities as shown for methionine synthase and aspartate transcarbamoylase while others don’t.

## Data Availability Statement

The raw data supporting the conclusions of this article will be made available by the authors, without undue reservation, to any qualified researcher.

## Author Contributions

AS and KB constructed strains and performed growth assays, western blots and enzyme assays. DB, FK, and SP analyzed protein abundance and acetylation. KB designed the study. KB and DB wrote the manuscript.

## Conflict of Interest

The authors declare that the research was conducted in the absence of any commercial or financial relationships that could be construed as a potential conflict of interest.

## Footnotes

1 http://www.matrixscience.com/

## References

[B1] Atsumi S. Cann A. F. Connor M. R. Shen C. R. Smith K. M. Brynildsen M. P. (2008). Metabolic engineering of *Escherichia coli* for 1-butanol production. *Metab. Eng.* 10 305–311. 10.1016/j.ymben.2007.08.003 17942358

[B2] Basan M. Hui S. Okano H. Zhang Z. Shen Y. Williamson J. R. (2015). Overflow metabolism in *Escherichia coli* results from efficient proteome allocation. *Nature* 528 99–104. 10.1038/nature15765 26632588 PMC4843128

[B3] Bernal V. Castaño-Cerezo S. Cánovas M. (2016). Acetate metabolism regulation in *Escherichia coli*: carbon overflow, pathogenicity, and beyond. *Appl. Microbiol. Biotechnol.* 100 8985–9001. 10.1007/s00253-016-7832-x 27645299

[B4] Blankenhorn D. Phillips J. (1999). Acid- and base-induced proteins during aerobic and anaerobic growth of *Escherichia coli* revealed by two-dimensional gel electrophoresis. *Society* 181 2209–2216. 10.1128/jb.181.7.2209-2216.1999 10094700 PMC93635

[B5] Bradford M. M. (1976). A Rapid and sensitive method for the quantitation of protein utilizing the principle of protein-dye binding. *Anal. Biochem.* 72 248–254. 10.1016/0003-2697(76)90527-3 942051

[B6] Brown T. D. Jones-Mortimer M. C. Kornberg H. L. (1977). The enzymic interconversion of acetate and acetyl-coenzyme A in *Escherichia coli*. *J. Gen. Microbiol.* 102 327–336. 10.1099/00221287-102-2-327 21941

[B7] Brunk E. Chang R. L. Xia J. Hefzi H. Yurkovich J. T. Kim D. (2018). Characterizing posttranslational modifications in prokaryotic metabolism using a multiscale workflow. *Proc. Natl. Acad. Sci. U.S.A.* 115 11096–11101. 10.1073/pnas.1811971115 30301795 PMC6205427

[B8] Carabetta V. J. Cristea I. M. (2017). The regulation, function, and detection of protein acetylation in bacteria. *J. Bacteriol.* 199 e107–e117. 10.1128/JB.00107-17 28439035 PMC5527388

[B9] Castaño-Cerezo S. Pastor J. M. Renilla S. Bernal V. Iborra J. L. Cánovas M. (2009). An insight into the role of phosphotransacetylase (pta) and the acetate/acetyl-CoA node in *Escherichia coli*. *Microb. Cell Fact.* 8 54. 10.1186/1475-2859-8-54 19852855 PMC2774668

[B10] Christensen D. G. Baumgartner J. T. Xie X. Jew K. M. Basisty N. Schilling B. (2019). Mechanisms, detection, and relevance of protein acetylation in prokaryotes. *MBio* 10 1–20. 10.1128/mBio.02708-18 30967470 PMC6456759

[B11] Contiero J. Beatty C. Kumari S. DeSanti C. L. Strohl W. R. Wolfe A. (2000). Effects of mutations in acetate metabolism on high-cell-density growth of *Escherichia coli*. *J. Ind. Microbiol. Biotechnol.* 24 421–430. 10.1038/sj.jim.7000014

[B12] Datsenko K. A. Wanner B. L. (2000). One-step inactivation of chromosomal genes in *Escherichia coli* K-12 using PCR products. *Proc. Natl. Acad. Sci. U.S.A.* 97 6640–6645. 10.1073/pnas.120163297 10829079 PMC18686

[B13] De Mey M. De Maeseneire S. Soetaert W. Vandamme E. (2007). Minimizing acetate formation in E. *coli fermentations*. *J. Ind. Microbiol. Biotechnol.* 34 689–700. 10.1007/s10295-007-0244-2 17668256

[B14] Diaz-Ricci J. C. Regan L. Bailey J. E. (1991). Effect of alteration of the acetic acid synthesis pathway on the fermentation pattern of *Escherichia coli*. *Biotechnol. Bioeng.* 38 1318–1324. 10.1002/bit.260381109 18600733

[B15] Dittrich C. R. Bennett G. N. San K.-Y. (2008). Characterization of the Acetate-Producing Pathways in *Escherichia coli*. *Biotechnol. Prog.* 21 1062–1067. 10.1021/bp050073s 16080684

[B16] Eiteman M. A. Altman E. (2006). Overcoming acetate in *Escherichia coli* recombinant protein fermentations. *Trends Biotechnol.* 24 530–536. 10.1016/j.tibtech.2006.09.001 16971006

[B17] el-Mansi E. M. T. Holms W. H. (1989). Control of carbon flux to acetate excretion during growth of *Escherichia coli* in batch and continuous cultures. *J. Gen. Microbiol* 135 2875–2883. 10.1099/00221287-135-11-2875 2693588

[B18] El-Mansi M. (2004). Flux to acetate and lactate excretions in industrial fermentations: physiological and biochemical implications. *J. Ind. Microbiol. Biotechnol.* 31 295–300. 10.1007/s10295-004-0149-2 15257440

[B19] El-Mansi M. (2005). Free CoA-mediated regulation of intermediary and central metabolism: An hypothesis which accounts for the excretion of α-ketoglutarate during aerobic growth of *Escherichia coli* on acetate. *Res. Microbiol.* 156 874–879. 10.1016/j.resmic.2005.04.008 16171983

[B20] Enjalbert B. Millard P. Dinclaux M. Portais J.-C. Létisse F. (2017). Acetate fluxes in *Escherichia coli* are determined by the thermodynamic control of the Pta-AckA pathway. *Sci. Rep.* 7:42135. 10.1038/srep42135 28186174 PMC5301487

[B21] Eydallin G. Ryall B. Maharjan R. Ferenci T. (2014). The nature of laboratory domestication changes in freshly isolated *Escherichia coli* strains. *Environ. Microbiol.* 16 813–828. 10.1111/1462-2920.12208 23889812

[B22] Feng J. Atkinson M. R. McCleary W. Stock J. B. Wanner B. L. Ninfa A. J. (1992). Role of phosphorylated metabolic intermediates in the regulation of glutamine synthetase synthesis in *Escherichia coli*. *J. Bacteriol.* 174 6061–6070. 10.1128/jb.174.19.6061-6070.1992 1356964 PMC207671

[B23] Förster A. H. Gescher J. (2014). Metabolic engineering of *Escherichia coli* for production of mixed-acid fermentation end products. *Front. Bioeng. Biotechnol.* 2:16. 10.3389/fbioe.2014.00016 25152889 PMC4126452

[B24] Fredericks C. E. Shibata S. Aizawa S.-I. I. Reimann S. A. Wolfe A. J. (2006). Acetyl phosphate-sensitive regulation of flagellar biogenesis and capsular biosynthesis depends on the Rcs phosphorelay. *Mol. Microbiol.* 61 734–747. 10.1111/j.1365-2958.2006.05260.x 16776655

[B25] Guest J. R. (1979). Anaerobic growth of *Escherichia coli* K12 with fumarate as terminal electron acceptor. genetic studies with menaquinone and fluoroacetate-resistant mutants. *J. Gen. Microbiol.* 115 259–271. 10.1086/330623 393800

[B26] Gupta S. Clark D. P. (1989). *Escherichia coli* derivatives lacking both alcohol dehydrogenase and phosphotransacetylase grow anaerobically by lactate fermentation. *J. Bacteriol.* 171 3650–3655. 10.1128/jb.171.7.3650-3655.1989 2661531 PMC210107

[B27] Han K. Lim H. C. Hong J. (1992). Acetic acid formation in *Escherichia coli* fermentation. *Biotechnol. Bioeng.* 39 663–671. 10.1002/bit.260390611 18600996

[B28] Harder B.-J. Bettenbrock K. Klamt S. (2016). Model-based metabolic engineering enables high yield itaconic acid production by *Escherichia coli*. *Metab. Eng.* 38 29–37. 10.1016/j.ymben.2016.05.008 27269589

[B29] Hellemans J. Mortier G. De Paepe A. Speleman F. Vandesompele J. (2007). qBase relative quantification framework and software for management and automated analysis of real-time quantitative PCR data. *Genome Biol.* 8:R19. 10.1186/gb-2007-8-2-r19 17291332 PMC1852402

[B30] Iwase T. Tajima A. Sugimoto S. Okuda K. I. Hironaka I. Kamata Y. (2013). A simple assay for measuring catalase activity: a visual approach. *Sci. Rep.* 3 3–6. 10.1038/srep03081 24170119 PMC3812649

[B31] Jenkins L. S. Nunn W. D. (1987). Regulation of the ato operon by the atoC gene in *Escherichia coli*. *J. Bacteriol.* 169 2096–2102. 10.1128/jb.169.5.2096-2102.1987 2883171 PMC212101

[B32] Kakuda H. Hosono K. Ichihara S. (1994). Identification and characterization of the ackA (Acetate Kinase A)-pta (Phosphotransacetylase) operon and complementation analysis of acetate utilization by an acka-pta deletion mutant of *Escherichia coli*. *J. Biochem.* 116 916–922. 10.1093/oxfordjournals.jbchem.a124616 7883769

[B33] Klein A. H. Shulla A. Reimann S. A. Keating D. H. Wolfe A. J. (2007). The intracellular concentration of acetyl phosphate in *Escherichia coli* Is sufficient for direct phosphorylation of two-component response regulators. *J. Bacteriol.* 189 5574–5581. 10.1128/JB.00564-07 17545286 PMC1951799

[B34] Kuhn M. L. Zemaitaitis B. Hu L. I. Sahu A. Sorensen D. Minasov G. (2014). Structural, kinetic and proteomic characterization of acetyl phosphate-dependent bacterial protein acetylation. *PLoS One* 9:e94816. 10.1371/journal.pone.0094816 24756028 PMC3995681

[B35] Laemmli U. K. (1970). Cleavage of structural proteins during the assembly of the head of bacteriophage T4. *Nature* 227 680–685. 10.1038/227680a0 5432063

[B36] Livak K. J. Schmittgen T. D. (2001). Analysis of relative gene expression data using real-time quantitative PCR and the 2(-Delta Delta C(T)) Method. *Methods San Diego Calif.* 25 402–408. 10.1006/meth.2001.1262 11846609

[B37] MacLean B. Tomazela D. M. Shulman N. Chambers M. Finney G. L. Chambers M. (2010). Skyline: an open source document editor for creating and analyzing targeted proteomics experiments. *Bioinformatics* 26 966–968. 10.1093/bioinformatics/btq054 20147306 PMC2844992

[B38] Majewski R. A. Domach M. M. (1990). Simple constrained-optimization view of acetate overflow in *E. coli*. *Biotechnol. Bioeng.* 35 732–738. 10.1002/bit.260350711 18592570

[B39] Martínez-Gómez K. Flores N. Castañeda H. M. Martínez-Batallar G. Hernández-Chávez G. Ramírez O. T. (2012). New insights into *Escherichia coli* metabolism: carbon scavenging, acetate metabolism and carbon recycling responses during growth on glycerol. *Microb. Cell Fact.* 11:46. 10.1186/1475-2859-11-46 22513097 PMC3390287

[B40] McCleary W. R. Stock J. B. (1994). Acetyl phosphate and the activation of two-component response regulators. *J. Biol. Chem.* 269 31567–31572. 7989325

[B41] McCleary W. R. Stock J. B. Ninfa A. J. (1993). Is acetyl phosphate a global signal in *Escherichia coli*Δ *J. Bacteriol.* 175 2793–2798. 10.1128/jb.175.10.2793-2798.1993 8491699 PMC204593

[B42] Molenaar D. van Berlo R. de Ridder D. Teusink B. Beg Q. Vazquez A. (2009). Shifts in growth strategies reflect tradeoffs in cellular economics. *Mol. Syst. Biol.* 5 931–942. 10.1038/msb.2009.82 19888218 PMC2795476

[B43] Nakayasu E. S. Burnet M. C. Walukiewicz H. E. Wilkins C. S. Shukla A. K. Brooks S. (2017). Ancient regulatory role of lysine acetylation in central metabolism. *MBio* 8 1–12. 10.1128/mBio.01894-17 29184018 PMC5705920

[B44] Oshima T. Aiba H. Masuda Y. Kanaya S. Sugiura M. Wanner B. L. (2002). Transcriptome analysis of all two-component regulatory system mutants of *Escherichia coli* K-12. *Mol. Microbiol.* 46 281–291. 10.1046/j.1365-2958.2002.03170.x 12366850

[B45] Paalme T. Elken R. Kahru A. Vanatalu K. Vilu R. (1997). The growth rate control in *Escherichia coli* at near to maximum growth rates: the A-stat approach. *Antonie Van Leeuwenhoek* 71 217–230. 9111915 10.1023/a:1000198404007

[B46] Pauli G. Overath P. (1972). ato operon: a highly inducible system for acetoacetate and butyrate degradation in *Escherichia coli*. *Eur. J. Biochem.* 29 553–562. 10.1111/j.1432-1033.1972.tb02021.x 4563344

[B47] Peebo K. Valgepea K. Maser A. Nahku R. Adamberg K. Vilu R. (2015). Proteome reallocation in *Escherichia coli* with increasing specific growth rate. *Mol. BioSyst.* 11 1184–1193. 10.1039/C4MB00721B 25712329

[B48] Pfeiffer T. Schuster S. Bonhoeffer S. (2001). Cooperation and competition in the evolution of ATP-producing pathways. *Science* 292 504–507. 10.1126/science.1058079 11283355

[B49] Pinhal S. Ropers D. Geiselmann J. De Jong H. (2019). Acetate metabolism and the inhibition of bacterial growth by acetate. *J. Bacteriol.* 201 1–19. 10.1128/JB.00147-19 30988035 PMC6560135

[B50] Prüß B. M. Wolfe A. J. (1994). Regulation of acetyl phosphate synthesis and degradation, and the control of flagellar expression in *Escherichia coli*. *Mol. Microbiol.* 12 973–984. 10.1111/j.1365-2958.1994.tb01085.x 7934904

[B51] Renilla S. Bernal V. Fuhrer T. Castaño-Cerezo S. Pastor J. M. Iborra J. L. (2012). Acetate scavenging activity in *Escherichia coli*: interplay of acetyl–CoA synthetase and the PEP–glyoxylate cycle in chemostat cultures. *Appl. Microbiol. Biotechnol.* 93 2109–2124. 10.1007/s00253-011-3536-4 21881893

[B52] Rossmann R. Sawers G. Böck A. (1991). Mechanism of regulation of the formate-hydrogenlyase pathway by oxygen, nitrate, and pH: definition of the formate regulon. *Mol. Microbiol.* 5 2807–2814. 10.1111/j.1365-2958.1991.tb01989.x 1779767

[B53] Sawers G. Böck A. (1988). Anaerobic regulation of pyruvate formate-lyase from *Escherichia coli* K-12. *J. Bacteriol.* 170 5330–5336. 10.1128/jb.170.11.5330-5336.1988 3053657 PMC211609

[B54] Sawers G. R. Clark D. P. (2004). Fermentative pyruvate and acetyl-coenzyme a metabolism. *EcoSal Plus* 1 10.1128/ecosalplus.3.5.3 26443368

[B55] Schilling B. Basisty N. Christensen D. G. Sorensen D. Orr J. S. Wolfe A. J. (2019). Global lysine acetylation in *Escherichia coli* results from growth conditions that favor acetate fermentation. *J. Bacteriol.* 201 1–9. 10.1128/jb.00768-18 30782634 PMC6456854

[B56] Schilling B. Christensen D. Davis R. Sahu A. K. Hu L. I. Walker-Peddakotla A. (2015). Protein acetylation dynamics in response to carbon overflow in *Escherichia coli*. *Mol. Microbiol* 98 847–863. 10.1111/mmi.13161 26264774 PMC4715485

[B57] Studdert C. A. Parkinson J. S. (2005). Insights into the organization and dynamics of bacterial chemoreceptor clusters through in vivo crosslinking studies. *Proc. Natl. Acad. Sci. U.S.A.* 102 15623–15628. 10.1073/pnas.0506040102 16230637 PMC1266109

[B58] Szenk M. Dill K. A. de Graff A. M. R. (2017). Why do fast-growing bacteria enter overflow metabolismΔ testing the membrane real estate hypothesis. *Cell Syst.* 5 95–104. 10.1016/j.cels.2017.06.005 28755958

[B59] Tanaka S. Lerner S. A. Lin E. C. (1967). Replacement of a phosphoenolpyruvate-dependent phosphotransferase by a nicotinamide adenine dinucleotide-linked dehydrogenase for the utilization of mannitol. *J. Bacteriol.* 93 642–648. 10.1128/jb.93.2.642-648.1967 4289962 PMC276489

[B60] Terol G. L. Jara J. G. Alba R. Martínez S. Díaz M. C. Puente T. D. D. (2019). Engineering protein production by rationally choosing a carbon and nitrogen source using E. *coli BL*21 acetate metabolism knockout strains. *Microb. Cell Fact* 18:151. 10.1186/s12934-019-1202-1 31484572 PMC6724240

[B61] Thakker C. Martínez I. San K.-Y. Bennett G. N. (2012). Succinate production in *Escherichia coli*. *Biotechnol. J.* 7 213–224. 10.1002/biot.201100061 21932253 PMC3517001

[B62] Theodorou M. C. Theodorou E. C. Kyriakidis D. A. (2012). Involvement of AtoSC two-component system in *Escherichia coli* flagellar regulon. *Amino Acids* 43 833–844. 10.1007/s00726-011-1140-7 22083893

[B63] Valgepea K. Adamberg K. Nahku R. Lahtvee P.-J. P. Arike L. Vilu R. (2010). Systems biology approach reveals that overflow metabolism of acetate in *Escherichia coli* is triggered by carbon catabolite repression of acetyl-CoA synthetase. *BMC Syst. Biol.* 4:166. 10.1186/1752-0509-4-166 21122111 PMC3014970

[B64] VanDrisse C. M. Escalante-Semerena J. C. (2019). Protein acetylation in bacteria. *Annu. Rev. Microbiol* 73 1–22. 31091420 10.1146/annurev-micro-020518-115526 PMC6736716

[B65] Varma A. Palsson B. O. (1994). Stoichiometric flux balance models quantitatively predict growth and metabolic by-product secretion in wild-type *Escherichia coli* W3110. *Appl. Environ. Microbiol.* 60 3724–3731. 10.1128/aem.60.10.3724-3731.1994 7986045 PMC201879

[B66] Veit A. Polen T. Wendisch V. F. (2007). Global gene expression analysis of glucose overflow metabolism in *Escherichia coli* and reduction of aerobic acetate formation. *Appl. Microbiol. Biotechnol.* 74 406–421. 10.1007/s00253-006-0680-3 17273855

[B67] Vemuri G. N. Altman E. Sangurdekar D. P. Khodursky A. B. Eiteman M. A. (2006). Overflow metabolism in *Escherichia coli* during steady-state growth: transcriptional regulation and effect of the redox ratio. *Appl. Environ. Microbiol.* 72 3653–3661. 10.1128/AEM.72.5.3653-3661.2006 16672514 PMC1472329

[B68] Wagner A. F. V. Schultz S. Bomke J. Pils T. Lehmann W. D. Knappe J. (2001). YfiD of *Escherichia coli* and Y06I of bacteriophage T4 as autonomous glycyl radical cofactors reconstituting the catalytic center of oxygen-fragmented pyruvate formate-lyase. *Biochem. Biophys. Res. Commun.* 285 456–462. 10.1006/bbrc.2001.5186 11444864

[B69] Wang J. Stieglitz K. A. Cardia J. P. Kantrowitz E. R. (2005). Structural basis for ordered substrate binding and cooperativity in aspartate transcarbamoylase. *Proc. Natl. Acad. Sci. U. S.A.* 102 8881–8886. 10.1073/pnas.0503742102 15951418 PMC1157055

[B70] Wanner B. L. (1992). Is cross regulation by phosphorylation of two-component response regulator proteins important in bacteriaΔ *J. Bacteriol.* 174 2053–2058. 10.1128/jb.174.7.2053-2058.1992 1551826 PMC205819

[B71] Weinert B. T. Iesmantavicius V. Wagner S. A. Schölz C. Gummesson B. Beli P. (2013). Acetyl-phosphate is a critical determinant of lysine acetylation in *E. coli*. *Mol. Cell* 51 265–272. 10.1016/j.molcel.2013.06.003 23830618

[B72] Wiśniewski J. R. Zougman A. Nagaraj N. Mann M. (2009). Universal sample preparation method for proteome analysis. *Nat. Methods* 6 359–362. 10.1038/nmeth.1322 19377485

[B73] Wolfe A. J. (2005). The acetate switch. *Microbiol. Mol. Biol. Rev.* 69 12–50. 10.1128/MMBR.69.1.12-50.2005 15755952 PMC1082793

[B74] Wolfe A. J. (2016). Bacterial protein acetylation: new discoveries unanswered questions. *Curr. Genet.* 62 335–341. 10.1007/s00294-015-0552-4 26660885 PMC4826803

[B75] Wolfe A. J. Chang D.-E. Walker J. D. Seitz-Partridge J. E. Vidaurri M. D. Lange C. F. (2003). Evidence that acetyl phosphate functions as a global signal during biofilm development. *Mol. Microbiol.* 48 977–988. 10.1046/j.1365-2958.2003.03457.x 12753190

[B76] Wyborn N. R. Messenger S. L. Henderson R. A. Sawers G. Roberts R. E. Attwood M. M. (2002). Expression of the *Escherichia coli* yfiD gene responds to intracellular pH and reduces the accumulation of acidic metabolic end products. *Microbiology* 148 1015–1026. 10.1099/00221287-148-4-1015 11932447

[B77] Yang Y.-T. Aristidou A. A. San K.-Y. Bennett G. N. (1999). Metabolic flux analysis of*Escherichia coli* deficient in the acetate production pathway and expressing thebacillus subtilis acetolactate synthase. *Metab. Eng.* 1 26–34. 10.1006/mben.1998.0103 10935752

[B78] Yu B. J. Kim J. A. Moon J. H. Ryu S. E. Pan J. G. (2008). The diversity of lysine-acetylated proteins in *Escherichia coli*. *J. Microbiol. Biotechnol.* 18 1529–1536. 18852508

[B79] Yun N.-R. San K.-Y. Bennett G. N. (2005). Enhancement of lactate and succinate formation in adhE or pta-ackA mutants of NADH dehydrogenase-deficient *Escherichia coli*. *J. Appl. Microbiol.* 99 1404–1412. 10.1111/j.1365-2672.2005.02724.x 16313413

[B80] Zhang K. Zheng S. Yang J. S. Chen Y. Cheng Z. (2013). Comprehensive profiling of protein lysine acetylation in *Escherichia coli*. *J. Proteome Res.* 12 844–851. 10.1021/pr300912q 23294111

[B81] Zhu J. Shalel-Levanon S. Bennett G. San K.-Y. (2007). The YfiD protein contributes to the pyruvate formate-lyase flux in an *Escherichia coli* arcA mutant strain. *Biotechnol. Bioeng.* 97 138–143. 10.1002/bit.21219 17013945

[B82] Zhuang K. Vemuri G. N. Mahadevan R. Beg Q. Vazquez A. Ernst J. (2014). Economics of membrane occupancy and respiro-fermentation. *Mol. Syst. Biol.* 7 500–500. 10.1038/msb.2011.34 21694717 PMC3159977

