# Supplementary material for: The Impact of ackA, pta, and ackA-pta Mutations on Growth, Gene Expression and Protein Acetylation in Escherichia coli K-12
Source: Front Microbiol. 2020 Feb 21;11:233. doi: 10.3389/fmicb.2020.00233 (PMC7047895; doi:10.3389/fmicb.2020.00233)
Supplement: Supplementary file 1 [file Data_Sheet_1.pdf]

Supplementary Material

1 Supplementary Tabale 1: Primers used for strain construction and Real Time qPCR

Primername	Sequence (5´....3´)	Protein
ackA-for-k.o+P1<br>ackA-rev-k.o+P2	TTTTTTAGCCACGTATCAATTATAGGTACTTCCatggtgtaggctggagctgcttcg<br>GCTGGCGGTGTGAAATCAGGCAGTCAGGCGGCTCGCcatatgaatatcctcctta	acetate kinase
pta-for-ko+P1<br>pta-rev-ko+P2	CGCCAAATCGGCGGTAACGAAAGAGGATAAACCGTGgtgtaggctggagctgcttcg<br>CGGATGATGACGAGATTACTGCTGCTGTGCAGACTGcatatgaatatcctcctta	phosphate acetyltransferase
ackA-for-NdeI<br>pta-rev-HindIII	aatcatatgatgTCGAGTAAGTTAGTACT<br>aataagcttGCGGATGATGACGAGATTAC	pRR-ackA-pta construction
pRR-ackA-pta-mut-1<br>pRR-ackA-pta-mut-2	TACAAAACAGCACCGCCAGCTGAGCTGGCGGTGTGAAATC<br>AAGCTTGGTACCTAACTAACT	pRR-ackA construciton
aceB-for-Real<br>aceB-rev-Real	GCGGCATGGCGGCGTTTATTC<br>GTGTCCGCAAGGCCTGGGTGAG	malate synthase A
aceE-For-Real<br>aceE-Rev-Real	ACGCGGCTGAAGGTAAAAACAT<br>CAGACACCGGCACATTGAAAC	pyruvate dehydrogenase complex
acs-For-Real<br>acs-Rev-Real	GCACCAGGCGGAAGAGATGAAC<br>AGCGCCGCGTACACCAGATAAC	acetyl-CoA synthetase
adhE-For-Real<br>adhE-Rev-Real	CACTCAATGGCGCACAAACTG<br>GCCTGCGGACGGTCATACTGG	alcohol dehydrogenase
arcA-for-real<br>arcA-rev-real	TGAGCTGAAACCGCACGAC<br>TCCAGATCACCGCAGAAGC	response regulator ArcA
crp-For-Real<br>crp-Rev-Real	CGAAAACCGCCTGTGAAGT<br>GACGACGCGCCATCTGTG	CRP transcriptional regulator
cydA-For-Real<br>cydA-Rev-Real	TGCGGCCTGTATACCCTGTTCC<br>CGTGCCGGCTGAGTAGTCGTG	terminal oxydase bd I
cyoA-For-Real<br>cyoA-Rev-Real	CCGCTGGCACACGACGAGA<br>AAGCGATTTCATTCACGGTAGCA	terminal oxidase bo

icdA-for-Real<br>icdA-rev-Real	ACCCGAACACTGGCAAAGAGA<br>TGCCAGGGCGTCAGAAATGTA	isocitrate dehydrogenase
ldhA-For-Real<br>ldhA-Rev-Real	TGCGCTGTGCCGGTTTCAATAA<br>CGGCGGTTCAGCGTCATCATC	lactate dehydrogenase
mgsA-for-Real<br>mgsA-rev-Real	TGGCAACGGCAGACTTCATAA<br>TCAGACGGTCCGCGAGATAAC	methylglyoxal synthase
ndh-for-Real<br>ndh-rev-Real	GTCGATCGTAACCACAGCCA<br>GCATGGGCCAGATAGCTCAA	NADH:quinone oxidoreductase II
nuoN-for-Real<br>nuoN-rev-Real	TGTCGCGTTGGGTAAAAACC<br>GAGAGAGTTTGAAGCCGAGGC	NADH:quinone oxidoreductase I
osmY-for-Real<br>osmY-rev-Real	TCGCGACGCTAAAGAAGG<br>GTACCGGAGAGCTGAACCAC	osmotically induced chaperone
pflB-For-Real<br>pflB-Rev-Real	GAGGCCCATACCACCGATAGAT<br>AGAAGCGCAGGAAATGGTTGAC	pyruvate-formate lyase
poxB-For-Real<br>poxB-Rev-Real	ATCATGCGCCACAACCAGTCGT<br>ACCGCGCAGGGCATGAACAATA	pyruvate oxidase
ptsG-For-Real<br>ptsG-Rev-Real	GGTTTTCCACGGCGACATTCC<br>GCGCGGTTTTCTGGTTTAGCA	PTS $EIICB^{Glc}$
recA-For-Real<br>recA-Rev-Real	CGCTTGGGGCAGGTGGTCT<br>TGCAGCGTCAGCGTGGTTTT	RecA
rpoD-for-Real<br>rpoD-rev-Real	TCTGCGTATGCGTTTCGGTATC<br>ACGGCTCGGGTGACGCAGTT	RNA Pol sigma 70
rpoS-for-Real<br>rpoS-rev-Real	AAAAATTGCCCGCCGTTATGG<br>AATCGCCCGTTCAATCGTCTG	RNA Pol sigma S
sdhA-For-Real<br>sdhA-Rev-Real	GAAAGGCGAAGATGTGGTTGT<br>CCCTGCTCGGCGATAGACTC	succinate dehydrogenase complex
sucA-For-Real,<br>sucA-Rev-Real	TCCGACACCGCGCAAAATCTAC<br>TCGGACGCCACTCTGCCACTAC	2-oxoglutarate decarboxylase complex
ybhC-for-Real<br>ybhC-rev-Real	GTCGCGGCGCAGTGGTGTT<br>ACGGCTGTTTACGGCGAGGAA	Ribosome maturation factor RimP

Supplementary Table 2: Growth data for mutant strains complemented with pRR-ackA-pta or pRR-ackA

	μ	Y_{ace}	Y_{suc}	Y_{EtOH}	Y_{Form}	Glc_up
	h^{-1}	mol/mol_{Glc}	mol/mol_{Glc}	mol/mol_{Glc}	mol/mol_{Glc}	mmol/(g *h)
**MG1655/pRR-ackA-pta**	0.35±0.07	0.82±0.1	0.12±0.02	0.67	1.72±0.2	12.2±0.6
**KBM1081/pRR-ackA-pta**	0.31±0.02	0.69±0.14	0.13±0.02	0.39	1.5±0.3	11.8±1.3
**KBM1082/pRR-ackA-pta**	0.37±0.04	0.66±0.26	0.12±0.024	0.46	1.28±0.11	15.9±4.2
**KBM1084/pRR-ackA-pta**	0.36±0.05	0.71±0.1	0.12±0.02	0.48	1.4±0.11	13.2±0.5
**MG1655/pRR-ackA**	0.46	0.8	0.11	0.79	1.81	13.8
**KBM1081/pRR-ackA**	0.47	0.74	0.14	0.61	1.5	14.4

Data represent mean data from two or three independent growth assays. For ethanol there are only data from one set of experiments. Data with plasmid pRR-ackA were performed only once.

Strains were grown as described under Material and methods. To select for plasmids pRR-ackA-pta and pRR-ackA 10 µg/ml ampicillin were added. No inducer was added to the cultures as induction led to severe growth inhibition in all strains.

Supplementary Table 3: Protein amounts significantly higher in KBM1081 vs MG1655 under anaerobic conditions

**Protein**	**KBM1081 [area]**	**MG1655 [area]**	**KBM1081 / MG1655 [ratio]**
Primosomal protein 1 OS=Escherichia coli (strain K12) OX=83333 GN=dnaT PE=1 SV=2	381721.9	0	
Citrate lyase acyl carrier protein OS=Escherichia coli (strain K12) OX=83333 GN=citD PE=2 SV=1	301175	0	
Uncharacterized electron transport protein YkgF OS=Escherichia coli (strain K12) OX=83333 GN=ykgF PE=3 SV=1	157805	0	
UPF0339 protein YegP OS=Escherichia coli (strain K12) OX=83333 GN=yegP PE=1 SV=2	51344.25	0	
Acetyl-CoA acetyltransferase OS=Escherichia coli (strain K12) OX=83333 GN=atoB PE=1 SV=1	11049383	929680.5	11.99
Zinc-binding GTPase YeiR OS=Escherichia coli (strain K12) OX=83333 GN=yeiR PE=1 SV=2	321412.5	55572.25	5.84
Acetate CoA-transferase subunit beta OS=Escherichia coli (strain K12) OX=83333 GN=atoA PE=1 SV=1	3283910	725408.5	4.57
Protein YfdX OS=Escherichia coli (strain K12) OX=83333 GN=yfdX PE=1 SV=1	2674433	758384	3.56
Acetate CoA-transferase subunit alpha OS=Escherichia coli (strain K12) OX=83333 GN=atoD PE=1 SV=1	3065906	898605	3.44
Ribosome-associated inhibitor A OS=Escherichia coli (strain K12) OX=83333 GN=raiA PE=1 SV=2	2020875	686322.5	2.97
UPF0381 protein YfcZ OS=Escherichia coli (strain K12) OX=83333 GN=yfcZ PE=3 SV=1	6702650	2400533	2.82
Formate acetyltransferase 1 OS=Escherichia coli (strain K12) OX=83333 GN=pflB PE=1 SV=2	9.61E+08	3.66E+08	2.65
Cystathionine gamma-synthase OS=Escherichia coli (strain K12) OX=83333 GN=metB PE=1 SV=1	18663510	7179317	2.62
ATP-dependent RNA helicase RhlE OS=Escherichia coli (strain K12) OX=83333 GN=rhlE PE=1 SV=3	225350	88621.5	2.57
Oxalyl-CoA decarboxylase OS=Escherichia coli (strain K12) OX=83333 GN=oxc PE=1 SV=1	3861790	1521166	2.56
Uncharacterized protein YmgD OS=Escherichia coli (strain K12) OX=83333 GN=ymgD PE=1 SV=2	11169115	4406599	2.56
UPF0337 protein YjbJ OS=Escherichia coli (strain K12) OX=83333 GN=yjbJ PE=1 SV=1	3023238	1278225	2.39

Homoserine O-succinyltransferase OS=Escherichia coli (strain K12) OX=83333 GN=metAS PE=1 SV=4	33755555	14560255	2.34
Uracil permease OS=Escherichia coli (strain K12) OX=83333 GN=uraA PE=1 SV=1	414231.8	178940	2.34
Formate hydrogenlyase subunit 7 OS=Escherichia coli (strain K12) OX=83333 GN=hycG PE=1 SV=2	981585	425320	2.33
Autonomous glycyl radical cofactor OS=Escherichia coli (strain K12) OX=83333 GN=grcA PE=1 SV=1	66790223	29243823	2.30
Chaperone protein ClpB OS=Escherichia coli (strain K12) OX=83333 GN=clpB PE=1 SV=1	1.94E+08	85016245	2.30
Protein ApaG OS=Escherichia coli (strain K12) OX=83333 GN=apaG PE=3 SV=1	235946.8	105622	2.25
Alcohol dehydrogenase YqhD OS=Escherichia coli (strain K12) OX=83333 GN=yqhD PE=1 SV=1	13594203	6123161	2.24
Uncharacterized protein YkgG OS=Escherichia coli (strain K12) OX=83333 GN=ykgG PE=3 SV=2	395043.3	180807.5	2.20
Dephospho-CoA kinase OS=Escherichia coli (strain K12) OX=83333 GN=coaE PE=1 SV=1	260265	119460	2.20
Branched-chain amino acid transport system 2 carrier protein OS=Escherichia coli (strain K12) OX=83333 GN=brnQ PE=1 SV=1	340850	158807.5	2.17
D-lactate dehydrogenase OS=Escherichia coli (strain K12) OX=83333 GN=ldhA PE=1 SV=1	17064392	8229834	2.09
RNA polymerase-associated protein RapA OS=Escherichia coli (strain K12) OX=83333 GN=rapA PE=1 SV=2	332877	161065	2.09
Protein YhjJ OS=Escherichia coli (strain K12) OX=83333 GN=yhjJ PE=1 SV=1	2796615	1354015	2.08
Biotin synthase OS=Escherichia coli (strain K12) OX=83333 GN=bioB PE=1 SV=2	767452.5	372975	2.08

Protein amounts were analyzed as described in Materials and Methods. Shown are the respective peak areas from MS analysis. For KBM1081 the total a total area of 19544628291 was detected and for MG1655 a total of 19719753850. Ratios were normalized to the total amount for each strain.

Supplementary Table 4: Protein amounts significantly lower in KBM1081 vs MG1655 under anaerobic conditions

Protein	KBM1081 [area]	MG1655 [area]	ratio KBM1081 / MG1655
Diguanylate cyclase DgcM OS=Escherichia coli (strain K12) OX=83333 GN=dgcM PE=1 SV=2	0	106730.3	
Vitamin B12 transporter BtuB OS=Escherichia coli (strain K12) OX=83333 GN=btuB PE=1 SV=2	0	85112.5	
HTH-type transcriptional regulator HexR OS=Escherichia coli (strain K12) OX=83333 GN=hexR PE=3 SV=2	0	70747.5	
Uncharacterized tRNA/rRNA methyltransferase LasT OS=Escherichia coli (strain K12) OX=83333 GN=lasT PE=3 SV=2	0	65053.25	
DNA helicase IV OS=Escherichia coli (strain K12) OX=83333 GN=helD PE=1 SV=2	0	53533	
Shikimate kinase 2 OS=Escherichia coli (strain K12) OX=83333 GN=aroL PE=1 SV=3	0	51237.75	
Acetate kinase OS=Escherichia coli (strain K12) OX=83333 GN=ackA PE=1 SV=1	3370957	79490623	0.04
Type-1 fimbrial protein, A chain OS=Escherichia coli (strain K12) OX=83333 GN=fimA PE=1 SV=2	261101.5	2485458	0.11
Uncharacterized HTH-type transcriptional regulator YafC OS=Escherichia coli (strain K12) OX=83333 GN=yafC PE=4 SV=1	290514.3	1648630	0.18
Inner membrane protein YagU OS=Escherichia coli (strain K12) OX=83333 GN=yagU PE=1 SV=1	1160090	6400425	0.18
Leu/Ile/Val-binding protein OS=Escherichia coli (strain K12) OX=83333 GN=livJ PE=1 SV=1	20737994	83876413	0.25
3-isopropylmalate dehydrogenase OS=Escherichia coli (strain K12) OX=83333 GN=leuB PE=1 SV=3	12605116	49459404	0.26
Cytosine permease OS=Escherichia coli (strain K12) OX=83333 GN=codB PE=1 SV=1	307903.5	1190463	0.26
NAD(P) transhydrogenase subunit alpha OS=Escherichia coli (strain K12) OX=83333 GN=pntA PE=1 SV=2	13176029	45219952	0.29
3-isopropylmalate dehydratase small subunit OS=Escherichia coli (strain K12) OX=83333 GN=leuD PE=1 SV=3	6872950	22344075	0.31
2-isopropylmalate synthase OS=Escherichia coli (strain K12) OX=83333 GN=leuA PE=1 SV=5	15314473	49350890	0.31

3-isopropylmalate dehydratase large subunit OS=Escherichia coli (strain K12) OX=83333 GN=leuC PE=1 SV=2	15918938	50308320	0.32
Glycine betaine/proline betaine transport system ATP-binding protein ProV OS=Escherichia coli (strain K12) OX=83333 GN=proV PE=1 SV=1	226942.8	716748.5	0.32
NAD(P) transhydrogenase subunit beta OS=Escherichia coli (strain K12) OX=83333 GN=pntB PE=1 SV=1	3568242	11056500	0.33
Chaperone protein FimC OS=Escherichia coli (strain K12) OX=83333 GN=fimC PE=1 SV=3	155845.3	476000	0.33
Pyrimidine-specific ribonucleoside hydrolase RihA OS=Escherichia coli (strain K12) OX=83333 GN=rihA PE=1 SV=2	528970	1591523	0.34
DNA-binding protein StpA OS=Escherichia coli (strain K12) OX=83333 GN=stpA PE=1 SV=1	4354240	12457360	0.35
Maf-like protein YhdE OS=Escherichia coli (strain K12) OX=83333 GN=yhdE PE=1 SV=1	138393.5	366222.5	0.38
30S ribosomal protein S19 OS=Escherichia coli (strain K12) OX=83333 GN=rpsS PE=1 SV=2	2473499	6234804	0.40
50S ribosomal protein L23 OS=Escherichia coli (strain K12) OX=83333 GN=rplW PE=1 SV=1	1300878	3140256	0.42
Dipeptide transport system permease protein DppB OS=Escherichia coli (strain K12) OX=83333 GN=dppB PE=1 SV=1	1023814	2462550	0.42
Formate hydrogenlyase subunit 3 OS=Escherichia coli (strain K12) OX=83333 GN=hycC PE=3 SV=2	82206	197655	0.42
Uncharacterized protein YbjX OS=Escherichia coli (strain K12) OX=83333 GN=ybjX PE=4 SV=1	85518.85	204137.5	0.42
DNA-binding protein Fis OS=Escherichia coli (strain K12) OX=83333 GN=fis PE=1 SV=1	206640	483902.5	0.43
Dipeptide transport ATP-binding protein DppF OS=Escherichia coli (strain K12) OX=83333 GN=dppF PE=3 SV=1	1976125	4595725	0.43
Leucine-specific-binding protein OS=Escherichia coli (strain K12) OX=83333 GN=livK PE=1 SV=2	5284119	12215609	0.44
Electron transport protein HydN OS=Escherichia coli (strain K12) OX=83333 GN=hydN PE=3 SV=1	543256.8	1251773	0.44
Hydrogenase-2 operon protein HybA OS=Escherichia coli (strain K12) OX=83333 GN=hybA PE=3 SV=1	51686.5	116528	0.45

Protein amounts were analyzed as described in Materials and Methods. Shown are the respective peak areas from MS analysis. For KBM1081 the total a total area of 19544628291 was detected and for MG1655 a total of 19719753850. Ratios were normalized to the total amount for each strain

Supplementary Table 5: RT-qPCR data for anaerobic growth.

	aceB	*aceE*	*pflB*	*acs*	*poxB*	*adhE*	*ldhA*
**KBM1081**	1.01 ±0.2	1.46 ±0.5	**3.76** ±1.3	1.86 ±0.4	**2.17 ±0.45**	1.14 ±0.1	**3.99** ±0.7
**KBM1082**	1.52 ±0.3	1.49 ±0.5	**2.52** ±0.9	1.26 ±0.1	1.37 ±0.2	0.92 ±0.1	**3.08** ±0.3
**KBM1084**	1.42 ±0.2	1.60 ±0.3	**3.80** ±1.9	1.37 ±0.2	0.87 ±0.1	0.87 ±0.1	**2.82** ±0.3

	ndh	*nuoN*	*cydA*	*cyoA*	*icdA*	*sdhA*	*sucA*
**KBM1081**	**1.91** ±0.5	1.11 ±0.7	1.50 ±0.1	**3.53** ±0.7	0.95 ±0.2	1.81 ±0.7	1.19 ±0.2
**KBM1082**	**2.47** ±1.0	1.24 ±0.2	1.29 ±0.1	1.57 ±0.4	0.95 ±0.2	1.03 ±0.1	0.95 ±0.1
**KBM1084**	**2.59** ±1.2	1.26 ±0.1	**2.18** ±0.1	1.99 ±0.6	1.09 ±0.2	1.32 ±0.1	0.98 ±0.1

	ppsA	*mgsA*	*pckA*	*ppc*	*osmY*	*arcA*	*crp*
**KBM1081**	**2.26** ±0.1	0.83 ±0.1	1.53 ±0.2	1.01 ±0.1	1.01 ±0.1	0.80 ±0.3	1.45 ±0.5
**KBM1082**	**1.99** ±0.1	0.87 ±0.1	1.80 ±0.1	0.76 ±0.1	1.20 ±0.2	0.70 ±0.1	1.27 ±0.3
**KBM1084**	**2.30** ±0.2	0.84 ±0.2	1.59 ±0.1	0.77 ±0.1	0.83 ±0.1	0.62 ±0.1	1.00 ±0.1

	rpoS	*ptsG*	*hycG*	*grcA*	*atoB*	*coaE*
**KBM1081**	0.63 ±0.2	**0.72** ±0.1	**0.12** ±0.01	**11.3** ±0.4	**15.7**±1.1	0.76 ±0.04

**KBM1082**	0.72 ±0.2	1.39 ±0.1	**0.06** ±0.001	**6.21** ±0.6	0.78 ±0.1	0.71 ±0.06
**KBM1084**	0.63 ±0.1	1.51 ±0.1	**0.06** ±0.001	**17.9** ±0.9	0.77 ±0.1	0.7 ±0.02

	recA	*ybhc*	*rpoD*
**KBM1081**	1.01 ±0.0	0.84 ±0.1	1.29 ±0.0
**KBM1082**	1.18 ±0.1	0.80 ±0.0	1.11 ±0.1
**KBM1084**	1.13 ±0.0	0.92 ±0.1	0.96 ±0.1

Gene expression was normalized to MG1655 grown at identical (anaerobic) conditions and to the housekeeping genes *recA*, *yhbC* and *rpoD*

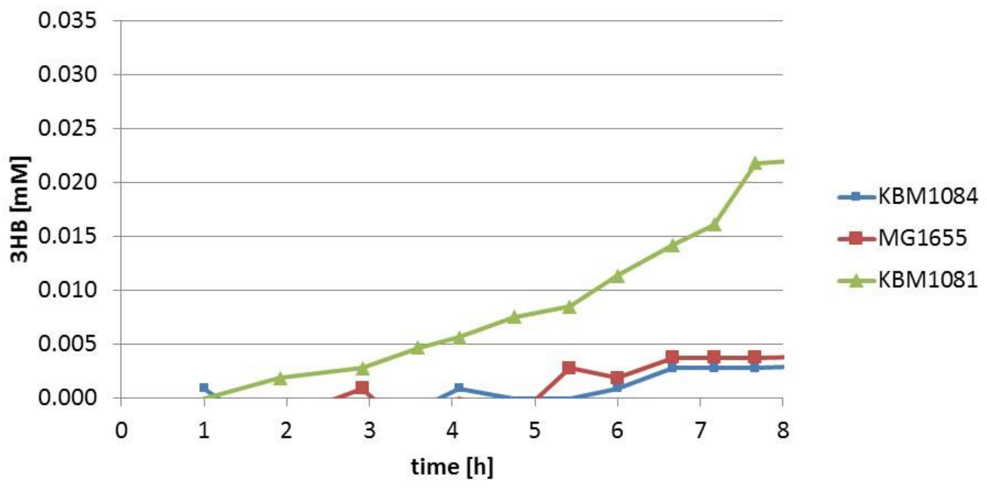

Supplementary Figure 1: 3HB production in KBM1081. Shown is data for extracellular 3HB concentrations from representative anaerobic growth curves of KBM1081 (*ackA*), KBM1084 (*ackA-pta*) and MG1655 growing in MM with 0.4 % glucose.

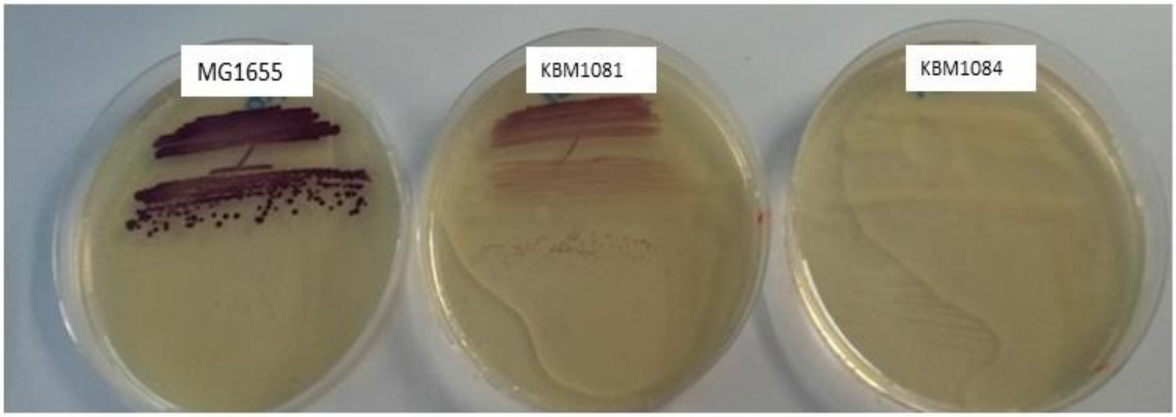

Supplementary Figure 2: PFL activity test. Strains were grown anaerobically on LB plates with 0.2% glucose and overlaid with softagar containing 1mg/ml benzylviologen and 100 mM pyruvate.

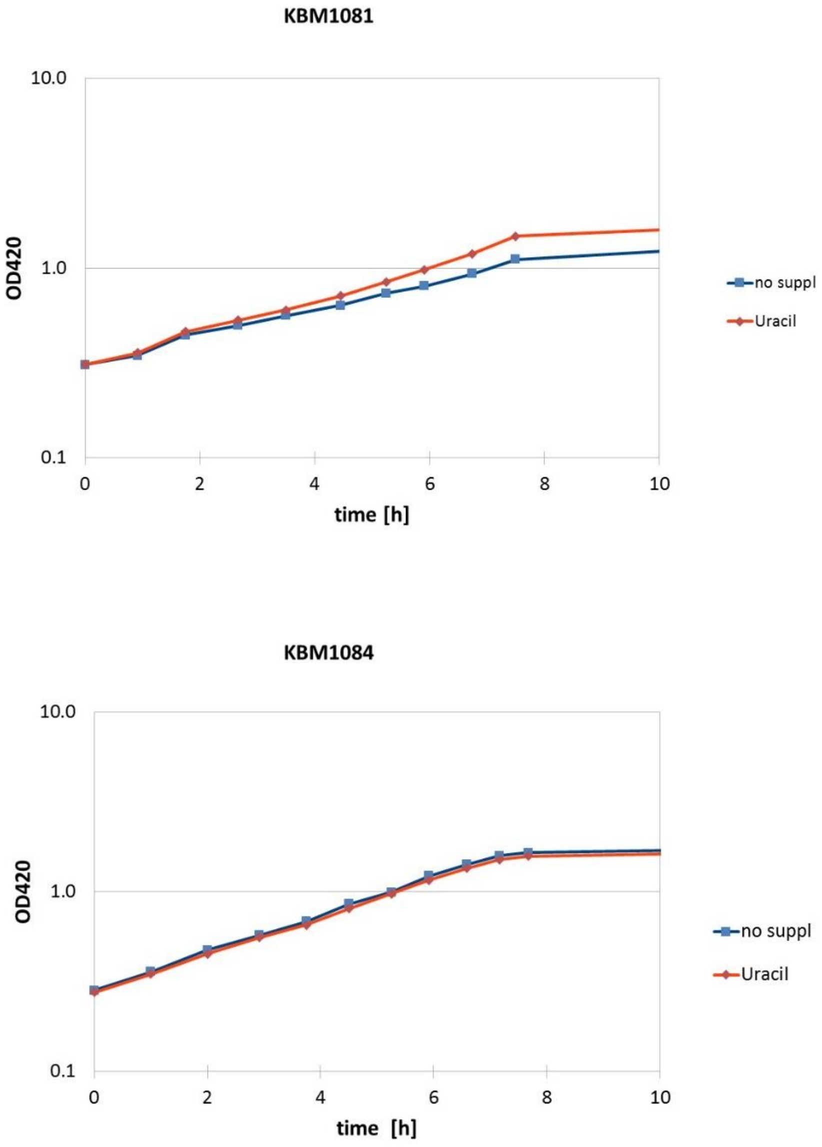

Supplementary Figure 3: Growth of KBM1081 and KBM1084 with uracil supplementation. Shown are representative growth curves of KBM1081 and KBM1084 growing in MM with 0.4 % glucose. To the cultures indicated 30µg/ml uracil was added 8red line).
